# Berry Polyphenols and Fibers Modulate Distinct Microbial Metabolic Functions and Gut Microbiota Enterotype-Like Clustering in Obese Mice

**DOI:** 10.3389/fmicb.2020.02032

**Published:** 2020-08-26

**Authors:** Maria-Carolina Rodríguez-Daza, Marcela Roquim, Stéphanie Dudonné, Geneviève Pilon, Emile Levy, André Marette, Denis Roy, Yves Desjardins

**Affiliations:** ^1^Institute of Nutrition and Functional Foods (INAF), Faculty of Agriculture and Food Sciences, Laval University, Québec, QC, Canada; ^2^Department of Food Science, Faculty of Agriculture and Food Sciences, Laval University, Québec, QC, Canada; ^3^Department of Plant Science, Faculty of Agriculture and Food Sciences, Laval University, Québec, QC, Canada; ^4^Department of Medicine, Faculty of Medicine, Cardiology Axis of Quebec Heart and Lung Institute, Laval University, Québec, QC, Canada

**Keywords:** *Akkermansia muciniphila*, *Coriobacteriaceae*, *Eggerthellaceae*, *Dubosiella*, fibers, polyphenols, flavonoids, prebiotics

## Abstract

Berries are rich in polyphenols and plant cell wall polysaccharides (fibers), including cellulose, hemicellulose, arabinans and arabino-xyloglucans rich pectin. Most of polyphenols and fibers are known to be poorly absorbed in the small intestine and reach the colon where they interact with the gut microbiota, conferring health benefits to the host. This study assessed the contribution of polyphenol-rich whole cranberry and blueberry fruit powders (CP and BP), and that of their fibrous fractions (CF and BF) on modulating the gut microbiota, the microbial functional profile and influencing metabolic disorders induced by high-fat high-sucrose (HFHS) diet for 8 weeks. Lean mice-associated taxa, including *Akkermansia muciniphila*, *Dubosiella newyorkensis*, and *Angelakisella*, were selectively induced by diet supplementation with polyphenol-rich CP and BP. Fiber-rich CF also triggered polyphenols-degrading families *Coriobacteriaceae* and *Eggerthellaceae*. Diet supplementation with polyphenol-rich CP, but not with its fiber-rich CF, reduced fat mass depots, body weight and energy efficiency in HFHS-fed mice. However, CF reduced liver triglycerides in HFHS-fed mice. Importantly, polyphenol-rich CP-diet normalized microbial functions to a level comparable to that of Chow-fed controls. Using multivariate association modeling, taxa and predicted functions distinguishing an obese phenotype from healthy controls and berry-treated mice were identified. The enterotype-like clustering analysis underlined the link between a long-term diet intake and the functional stratification of the gut microbiota. The supplementation of a HFHS-diet with polyphenol-rich CP drove mice gut microbiota from *Firmicutes/Ruminococcus* enterotype into an enterotype linked to healthier host status, which is *Prevotella/Akkermansiaceae*. This study highlights the prebiotic role of polyphenols, and their contribution to the compositional and functional modulation of the gut microbiota, counteracting obesity.

## Introduction

Lower microbial diversity and altered metabolic functions in the gut microbiota (i.e., dysbiosis), is conducive to many health problems and is particularly linked to the development of obesity (Turnbaugh et al., 2006, 2008). Indeed, dysbiotic fecal microbiota transfer from obese donors into lean germ-free mice reproduces obesity phenotype and metabolic alterations in the latter (Turnbaugh et al., 2008). The link between the gut microbiome, obesity and diet, has been as well established by enterotypes functional stratification of gut microbes while accounting for their capacity to use unabsorbed nutrients, thus, affecting the host fitness (Nakayama et al., 2017; Costea et al., 2018). Therefore, identifying microbes conferring metabolic benefits is essential to develop dietary strategies favoring their activity in order to maintain or restore health.

In recent years, prebiotics have gained interest for alleviating gut dysbiosis and decreasing the risk of metabolic disorders (Sanders et al., 2019). Prebiotics are non-digestible food ingredients selectively stimulating the growth and activity of a limited number of colonic bacteria, beneficially impacting host health (Gibson et al., 2017). Prebiotic polysaccharides, such as pectin, arabinoxylans, cellulose and hemicellulose, favor the growth of *Bifidobacterium* spp., *Lactobacillus* spp., *Faecalibacterium prausnitzii*, *Bacteroides* spp., and *Prevotella* spp. exerting protective effects against metabolic diseases (Chen et al., 2018; Larsen et al., 2019; Mao et al., 2019). Cellulose and hemicellulose, besides arabinans and arabino-xyloglucans rich pectin, are components of cranberry cell walls, constituting 65.5% insoluble fiber of whole fruit (White et al., 2010; Hotchkiss et al., 2015). Likewise, blueberry cell wall hemicellulose is rich in pectin and xyloglucan (30–35%) (Vicente et al., 2007; Blaker and Olmstead, 2015). These prebiotic fibers are fermented to short chain fatty acids (SCFAs) by the gut microbiota, and are involved in intestinal cell turnover, lipid metabolism, and glycemic response (De Vadder et al., 2014; Makki et al., 2018). Furthermore, plant cell wall components can interact with polyphenols, such as proanthocyanins (PACs), as encountered in apple, cranberry and grapes (Bourvellec et al., 2005; Bindon et al., 2010; Hotchkiss et al., 2015). Tightly linked to fibers, polyphenols are catabolized by gut microbes to smaller phenolic acids, which exert anti-inflammatory and immuno-modulatory effects in the host (González-Sarrías et al., 2017).

Polyphenols also exert prebiotic effects and reduce the risk of obesity, insulin resistance, and cardiovascular diseases (Skrovankova et al., 2015; den Abbeele et al., 2018). Between 90 and 95% of ingested polyphenols reach the colon, where they suppress pathobionts and stimulate health promoting species (Clifford, 2004; Masumoto et al., 2016). For instance, diet enrichment with whole wild blueberries increased symbiotic microbes of *Bifidobacteriaceae* and polyphenol-degrading family *Coriobacteriaceae* in rodent and human guts (Guglielmetti et al., 2013; Lacombe et al., 2013a; Rodríguez-Daza et al., 2020). Likewise, cranberry polyphenols and seed fibers inhibited pathobionts and stimulated the probiotics *Bacillus coagulans* and *Lactobacillus rhamnosus* (Lacombe et al., 2013b; Majeed et al., 2018). Importantly, several polyphenolic extracts concomitantly inhibited opportunistic pathogens and stimulated *Akkermansia muciniphila* in mice fed an obesogenic diet (Anhê et al., 2014; Collins et al., 2016; Tu et al., 2018; Rodríguez-Daza et al., 2020). This bacterium is of a particular interest due to its immuno-modulatory properties and its capacity to prevent obesity, type-2 diabetes and host inflammation (Everard et al., 2013; Schneeberger et al., 2015; Ansaldo et al., 2019). Even if many evidences point to the prebiotic effects of both berry fibers and polyphenols, more work is needed to differentiate the role of cell wall polysaccharides from that of polyphenols on gut microbiota composition and function.

The present study aims to evaluate the prebiotic potential of cranberry and blueberry whole fruit powders and that of their fibrous fractions (non-extractable polyphenols and polysaccharides) on gut microbiota composition and functional profile, in a murine model of high-fat high-sucrose (HFHS)-induced obesity. Polyphenol-rich whole berry fruit powders selectively favored key species linked to lean phenotypes and attenuation of obesity-related disorders. Changes in the predicted functional profile and metabolic redundancy of gut microbiota reveal a distinct role of polyphenol-rich cranberry powder in the prevention of obesity-associated phenotypes. While the whole blueberry powder modulated the gut microbiota composition, it did not significantly improve microbial functions.

## Materials and Methods

### Berry Powders and Fibers Characterization

American cranberry (*Vaccinium macrocarpon* Aiton) and wild blueberry (*Vaccinium angustifolium* Aiton) fruit powders were provided by Fruits d’Or (Quebec, Canada) and fibrous residues were provided by Diana Food Canada. The fruit fibers were obtained after extraction of polyphenols with a 70% hydroethanolic extract as described previously (Denis et al., 2015). The extraction solid residues were pressed and air dried at 50°C and powdered before inclusion in the mice feed. The phenolic characterization of the fruit powders and fibrous residues was carried out according to the methodology described by Dudonné et al. (2015). Briefly, the total phenolic content was measured by the Folin-Ciocalteu technique by quantifying on the basis of gallic acid equivalent (Sigma-Aldrich, St. Louis, MO, United States). The anthocyanins were analyzed by reverse phase HPLC with a diode array detector and quantified using a cyanidin 3-glycoside standard (Extrasynthèses, Genay, France). Proanthocyanidins were separated according to their degree of polymerization (DP) by normal phase HPLC chromatography and quantized by fluorescence using (-)-epicatechin as standard (Sigma-Aldrich, St. Louis, MO, United States). Chromatography grade solvents were purchased from EMD Millipore Chemicals (Billerica, MA, United States) and Anachemia (Montreal, Canada).

A comprehensive analysis of the fibers, proteins, lipids, carbohydrates and total energy content of the samples was conducted by Environex (Quebec, Canada) using standard AOAC methods for total lipids (AOAC 989.05), for proteins (AOAC 992.15), for dietary fibers (AOAC 985.29), and moisture content (AOAC 44-15A). Caloric and carbohydrate contents were calculated. The carbohydrate content was obtained by subtracting the dietary fibers content from the total carbohydrate content.

### Diet Preparation

Cranberry and blueberry powders and their fibrous residue have been uniformly incorporated into a HFHS. The amount of polyphenol-rich cranberry (CP) and blueberry powders (BP) was defined on the basis of the polyphenol dose of 200 mg/kg of body weight (BW) in mice, as previously determined (Anhê et al., 2014). Subsequently, the amount of cranberry and blueberry fiber-rich fractions (CF and BF, respectively) was calculated considering the equivalent total fiber content as found in the corresponding berry powders (see Table 1). To maintain an isocaloric level, the diets composition was adjusted to provide equivalent nutritional value of 5.4 kcal/g.

**TABLE 1:** Diets adjustment with berry powders and fibrous fractions.

Ingredients (g/100 g)	HFHS	HFHS + Cranberry powder		HFHS + Cranberry fiber		HFHS + Blueberry powder		HFHS + Blueberry fiber	
Protein (Casein/L-cystine)	20.18	20.04	0.14	19.84	0.35	20.06	0.12	20.06	0.12
Carbohydrate (sucrose)	26.89	21.03	5.84	26.89	–	23.05	3.84	26.83	0.06
Fibers (Cellulose/berry’s fibers)	5	2.5	2.5	2.6	2.4	4.2	0.8	4.2	0.8
Fat (lard/corn oil)	39.6	38.53	1.1	38.7	0.85	39.43	0.17	39.54	0.06
Mineral mix	6.7	6.7	-	6.7	–	6.7	–	6.7	–
Vitamin mix	1.4	1.4	-	1.4	–	1.4		1.4	–
Choline bitartrate	0.2	0.2	-	0.2	–	0.2	–	0.2	–
Tert-butylhydrytoluene (BHT)	0.03	0.03	-	0.03	–	0.03	–	0.03	–
Residual humidity + ashes	–	–	0.4	–	0.25	–	0.1	–	0.12
Total (g)	100	100.41		100.21		100.13		100.12	
Energetic density (kcal/g)	5.45	5.42		5.43		5.44		5.44	

### Experimental Design of Animal Bioassay

Six-weeks-old C57BL/6J male mice (*n* = 72; Jackson laboratories, Sacramento, CA, United States) were randomized into six groups of 12 animals each and were single-housed in a controlled environment (one mouse per cage; 12/12-h light-dark) with free access to food and drinking water. After 2 weeks of acclimation with standard-chow diet (2018 Teklad global 18% protein rodent diet, Harlan laboratories), the mice were fed either a Chow-diet (CT), a HFHS control diet or a HFHS diet containing either polyphenol-rich CP and BP, or the fibrous fractions CF and BF for 8 weeks (see Table 1). BW gain was recorded under non-fasting condition, twice a week. Food intake was evaluated by monitoring the food consumption (g) three times per week. After 5 and 7 weeks of treatment, insulin tolerance test (ITT) and oral glucose tolerance test (OGTT) were performed, respectively. Fecal material was collected from each mouse for bacterial DNA extraction before the start of the treatments (week 0) and at the end (week 8), immediately immersed in dry-ice and stored at −80°C. After 8 weeks of feeding, mice were anesthetized with isoflurane (2–3%; 0.5–1.5 L/min) and euthanized by cardiac puncture. Adipose tissues, organs, and intestines were carefully dissected; their weight was recorded and immediately immersed in liquid nitrogen, RNA later (Invitrogen) or into fixation solutions (Carnoy’s solution, buffered formalin 10%) according to the subsequent analysis. Frozen tissues were then stored at −80°C. All experimental procedures were performed according to the guidelines of the animal care committee of Laval University (CPAUL). The protocols were summited and approved by Canadian Council on Animal Care.

### Metabolic Phenotypes Assessment

After 5 weeks of treatments, mice were fasted for 6 h. Blood was drawn at baseline and immediately centrifuged (3,500 rpm, 10 min at 4°C). Insulin solution was administered 10 μL/g of 0.65 U/kg by intraperitoneal injection, followed by glycemia measurements using an Accu-Check glucometer (Bayer) before and at 5, 10, 15, 20, 30, and 60 min after the injection. An OGTT was performed after 7 weeks of treatment. Mice were fasted for 12 h overnight, blood baseline sample was drawn and immediately centrifuged (3,500 rpm, 10 min at 4°C). Dextrose solution (2 μL/g of 50% dextrose) was administered by gavage and blood samples (60 μL) were obtained from the mouse lateral saphenous vein. Glycemia was determined using an Accu-Check glucometer (Bayer) at time 15, 30, 60, 90, and 120 min relative to the glucose load. The area under the curve (AUC-OGTT) was determined considering the glucose levels measured between baseline and 120 min after glucose overload (GraphPad Prism 7.0, United States). Insulin resistance index (HOMA-IR) was calculated using the formula: HOMA-IR = [fasting glycemia (nmol/L) × fasting insulinemia (μU/mL)/22.5]. The 12 h fasting blood samples were collected for determinations of plasma insulin. Samples were stored at −80°C until the assay. Mouse insulin was determined using an ultrasensitive ELISA kit (Alpco, Salem, United States).

Liver triglyceride (TG) content was assessed after chloroform–methanol extraction and enzymatic reactions using commercial kits (Randox Laboratories, Crumlin, United States).

### Colon Histomorphology

Colon tissues were prepared as previously described (Williams et al., 2016). The PBS-flushed colon tissues were cut into two equal-sized sections (transverse incision), and then were transferred into the tube containing a large excess of cold Methanol-Carnoy’s solution (60% methanol, 30% chloroform, and 10% glacial acetic acid). The samples were stored for 3 h at 4°C to allow the fixation. After fixation, the tissues were washed with cold 70% ethanol once, and stored in the same solution at 4°C until they were processed. Tissue processing was performed at IBIS Laboratory of Molecular Imaging and Microscopy, Laval University (Québec, QC, Canada). The samples were placed into a tissue cassette indelibly labeled with a unique animal identifier. The cassettes were subjected to a tissue processor for standard processing to allow embedding in paraffin wax (Tissue-Tek VIP, vacuum Infiltration Processor Sakura brand). The protocol applied was: Alcohol 95% 45 min, Alcohol 100% 3 × 45 min, Toluene 2 × 45 min, Paraffin 1h30, 2h00, and 4h00. Paraffin-embedded sections of colon tissues were cut and stained with both periodic acid Schiff and Alcian blue (AB-PAS). AB-PAS staining enables the differentiation between acid mucins and neutral mucins, with blue color showing acid mucins, purple color indicating a mixture of neutral and acid mucins, and red/magenta color indicating neutral mucins alone (Barcelo et al., 2000).

### Goblet Cell Quantification, Mucus Thickness and Crypts Depth Measurements

The relative proportions and distribution of mucous secreting goblet cells (GC) were quantified following Johnson et al. (2016) protocols with some modifications (Johansson et al., 2014; Johnson et al., 2016). All images were captured using a BX51 microscope (Olympus America, Inc., United States) equipped with a CCD Digital Camera System and Image-Pro software. Number of GC was counted for a defined distance (using 20× objective lens, 50 μm scale). Initially, images were taken with the 4× objective lens (200 μm scale bars) in order to examine the whole tissue section. This was used to ensure that the total area of the images taken at a higher magnification would cover at least 50% of the tissue section. Afterward, under 20× objective lens, each section was divided into four equal quadrants and taking representative images. A total of 12 crypts (three crypts per section) were analyzed for each mouse. In each quadrant, within a delineated area (from epithelium toward the colonic crypts), mucin secreting GC types (acid, neutral and mixed) were counted and the data were expressed as the relative cell numbers per crypt (12 crypts of 12 mice per group). To determine the specific mucin GC types for the colon for each animal, the cell counts were totaled separately in acid, neutral and mixed mucin-filled GC, and the data normalized to reflect the total numbers of each mucin cell type per crypt. Crypt lengths (μm) were calculated from an average of 12 crypts per tissue section, for 12 mice. Average of mucus thickness was calculated in a similar manner, measuring the lengths of the inner mucus layer closely adhered to the epithelium, for 12 mice. The National Institute of Health image J software was used for supporting the cell quantification and crypts length measurements.

### Fecal DNA Extraction and 16S rRNA Sequencing

The microbial genomic DNA was extracted from approximately 100 mg of feces using a ZR fecal DNA kit (D6010; Zymo Research Corp., Orange, CA, United States) following the manufacturer protocols. The concentration and quality of DNA was determined spectrophotometrically using a ND-1000 Nanodrop (Nanodrop Technologies, Wilmington, DE, United States).

Degenerate primers 341F (5′-CCTACGGGNGGCWGCAG-3′) and 805R (5′-GACTACHVGGGTATCTAATCC-3′) targeting the 16S rRNA V3–V4 region were used for the amplicon library preparation of 16S rRNA fragments. The primers were adapted to incorporate the transposon-based Illumina Nextera adapters (Illumina, United States) and a sample barcode sequence allowing multiplexed paired-end sequencing. Constructed 16S metagenomic libraries were purified using 35 μL of magnetic beads (AxyPrep Mag PCR Clean up kit; Axygen Biosciences, United States) per 50 μL PCR reaction. Library quality control was performed with a Bioanalyzer 2100 using DNA 7500 chips (Agilent Technologies, United States). An equimolar pool was obtained and checked for quality prior to further processing. The pool was quantified using picogreen (Life Technologies, United States) and loaded on a MiSeq platform using 2 × 300 bp paired-end sequencing (Illumina, United States). High-throughput sequencing was performed at the IBIS (Institut de Biologie Intégrative et des Systèmes – Université Laval).

### 16S rRNA Sequences Processing

Demultiplexed RAW data files covering all of the samples were imported into the R studio environment (version 3.6.1, R Core Team, Vienna, Austria). Paired forward and reverse reads from raw data files were trimmed (primer removal) using Cutadapt (version 2.4) (Marcel, 2011). Reads were quality-filtered and bases with low-quality scores (<20) were discarded. These filtered files were then processed using Divisive Amplicon Denoising Algorithm (DADA2) pipeline which included the steps of dereplication, core denoising algorithm (that models and corrects Illumina-sequenced amplicon errors) and the merging of the base pairs. The advantage of using DADA2 over traditional clustering methods is that it determines exact sequences based on an error model for the sequencing run, resolving differences of as little as one nucleotide (Callahan et al., 2016), merging function provided global ends-free alignment between paired forward and reverse reads. The reads were merged together if they overlapped exactly, and a table for amplicon sequence variants (ASVs, a higher analog of operational taxonomic units – OTUs) was constructed, recording the number of times each ASV was observed in each sample. Chimeras were removed using the *removeBimeraDenovo* function of the same DADA2 package. ASVs sequences were assigned a taxonomy using the most recent SILVA taxonomic database (SILVA SSU Ref 132 NR, Dec 2017) as reference dataset (Quast et al., 2013). A phylogenetic tree of the ASVs was obtained using the function *AlignSeq* implemented in DECIPHER R package (Wright, 2016) to create the multiple sequence alignment used to inform downstream analyses in *phyloseq* package, especially the calculation of phylogeny-aware distances between microbial communities. Afterward, a phyloseq data object was created using the *phyloseq* package in R (McMurdie and Holmes, 2013). Unassigned taxa and singletons were removed. Estimates of observed α-diversity, richness (Chao1) and diversity (Shannon and Simpson Indices) were measured within sample categories using *estimate_richness* function of the *phyloseq* package. Relative abundances of microbial genera and phylum were plotted using the *ggplot2* packag (Wickham, 2009) after transforming abundance data into relative abundances. For β-diversity analysis, multidimensional scaling (MDS; also known as principal coordinate analysis, PCoA) was performed while using different distance measures: the Bray–Curtis dissimilarity that considers the species abundance count and the unweighted UniFrac that accounts for phylogenetic closeness of ASVs (OTUs) observed in different samples without taking into account the abundances. The plots of community data were visualized by using their base functions in the *phyloseq* package.

### Microbial Functional Profiling

To evaluate whether the cranberry and blueberry powders or their fiber fractions drive microbes with distinct functional repertoires, we investigated the functional profile of each ASV inferred against SILVA database. Predicted functions of each sample were extracted based on the Kyoto Encyclopedia of Genes and Genomes (KEGG) Ontology (KO) functional and pathway profiles using Tax4Fun2 (Wemheuer et al., 2018). A current default reference dataset of 16S rRNA gene data from 12,002 bacterial genomes (NCBI RefSeq database assessed on 19 August, 2018) is available through the KEGG database to generate reference data (Wemheuer et al., 2018). The ASV count table and the taxonomic classification were merged and then, used to predict functional profiles. ASV fasta file were initially aligned against the supplied 16S rRNA reference sequences (“Ref100NR”) with copy number correction enabled by BLAST (version 2.9.0), using the *runRefBlast* function. Functional predictions were subsequently calculated using the *makeFunctionalPrediction* function incorporating the abundance of each ASV. Only ASVs that were matched with a 97% similarity to sequences present in the SILVA database were included. ASVs assigned to a taxonomic key (i.e., a particular genus) having no functional reference profiles were not included in the prediction (called fraction of unexplained, FTU). The obtained profile is subsequently normalized: the sum of all functions in a sample is one. The functional redundancy index (FRI) with respect to single functions was also calculated. The FRI denotes the proportion of species capable of performing a particular function and their phylogenetic relationship to each other. A specific function is almost ubiquitous in all community members when there is a high FRI, whereas functions that are present in a few closely related species or that have been detected in only one community member is indicated by a low FRI (Wemheuer et al., 2018). We subsequently compared these FRI values based on the actual genomic information of the observe bacterial abundance changes by calculating the Bray–Curtis distances and analysis of similarities (PERMANOVA); principal coordinate analyses were performed to visualize the closeness of functions displayed by bacterial communities making up the gut microbiota of each group.

### Multivariate Association Analysis

Multivariate microbial Association with Linear Models (MaAsLin) was performed for overlapping the diet composition (polyphenol-containing diet, the fiber-rich diets and the control diets) as covariates, while accounting for bacterial taxonomies (at the genus level), potentially determining the significance to a specific variable. The MaAsLin system uses an arcsine square root transformed analysis of the microbial community abundance or function in a standard multivariable linear model, pertinent to determine the significance of putative relationships from clinical or environmental metadata set (Mallick et al., 2019). Using the MaAsLin model, the analysis was focused on the diet composition as a single variable of interest, “subtracting out” the effect of the other confounding metadata variables evaluated in the present study. The minimum percentage relative abundance of 0.01% was applied within the MaAsLin parameters, *p* values across all associations were then adjusted using the Benjamini–Hochberg false discovery rate (FDR) method, with a threshold of *p* < 0.05 and *q* < 0.05. Only the microbial features with *q* < 0.05 were reported.

To evaluate the association of the gut microbiota functional profiling with changes in the liver TG, the energetic efficiency and BW, “MaAslin” was run with same settings across all. Then, functional pathways at the KEGG level 1 from Tax4Fun2 data were included. The functional features presented were representatives pathways that were significantly modulated by the diets as determined by LEfSe analysis (*q* < 0.05) (Segata et al., 2011).

Likewise, all the covariates such as BW gain, liver TG, inguinal white adipose tissue (IWAT), epididymal white adipose tissues mass (EWAT), energetic efficiency, cecum weight and mucus thickness were also analyzed to look for significant changes that could be driven by specific bacterial taxa during the 8-weeks dietary intervention. The co-occurrence relationships between the relative abundances of taxa (at the genus level) found to be significantly associated with the above variables were evaluated by calculating Spearman ranked correlations. The resulting association were visualized using a heatmap representing the R correlation coefficients. In the heatmap, the *p* values across all associations are presented as ^∗^*p* < 0.05, ^∗∗^*p* < 0.01, ^∗∗∗^*p* < 0.001, and ^****^p < 0.0001. Relations between bacterial communities and mouse phenotypes of the different groups of mice were further represented using Ordination of Canonical Correspondence Analysis (CCA). CCA allows exploration of relations between sets of variables – in this case, microbiota composition according to the diet and the mouse phenotypes found with significant changes during the intervention. CCA strives to maximize the correlations of the gut microbiota structure with one set of variables while accounting for the microbiota composition and the type of diet. CCAs were computed using R studio and *Vegan* package.

### Gut Microbiota Enterotype-Like Clustering

Functional stratification linking the mice gut microbiota to the 8-week dietary supplementation was performed according to the enterotyping approach^1^ in R as previously described by Arumugam et al. (2011) and Costea et al. (2018). Briefly, Jensen-Shannon divergence (Calinski and Harabasz, 1974) of genera abundance table of mouse gut microbiota (*n* = 12 per dietary group) was calculated in R. This distance matrix of β-diversity was submitted to partitioning around medoids (PAM) clustering by using *Cluster* and *ClusterSim* R packages. The optimal cluster number was determined by the Calinski–Harabasz index (Calinski and Harabasz, 1974) with the “*index.G1()*” function from *clusterSim* R package (Walesiak, 2008), and then, they were validated by calculating the silhouette index (Rousseeuw, 1987) by using “*silhouette()*” function from *cluster* R package (Maechler et al., 2019). The result of enterotype-clustering was visualized in a PCoA plot using “*s.class*” function from *ade4* R package (Dray and Dufour, 2007).

### *A. muciniphila* Quantification by qPCR

The determination of 16S rRNA gene copies of total bacteria was carried out per sample through quantitative PCR amplification using the bacterial universal primers Uni334F (5′-ACTCCTACGGGAGGCAGCAGT-3′) and Uni514R (5′-ATTACCGCGGCTGCTGGC-3′) (Hartman et al., 2009). The proportion of *A. muciniphila* 16S rRNA gene copy relative to the total bacteria was analyzed in fecal samples from all groups. Home designed 16S rRNA primer sequences (Geneious software, version 9.0) were used for *A. muciniphila* (forward 5′-CACACCGCCCGTCACAT-3′ and reverse 5′-TGCGGTTGGCTTCAGATACTT-3′). Primer amplification efficiency and standard curves were determined by making dilution series of pure total DNA for this bacterium (ATCC BAA-835 strain), calculating a linear regression based on the CT data points, and inferring the efficiency from the slope of the line. The reaction mixture (20 μL) contained 10 μL of 2× PowerUP SYBR Green Master mix (Applied Biosystem), 6.4 μL of water, 0.8 μL of a 5 μM Forward and Reverse primers, and 2 μL of extracted DNA in DNase/RNase-free water. The qPCR amplifications were performed on an Applied Biosystems ABI 7500 Fast real-time cycling platform. The thermocycling protocol consisted in 50°C for 2 min, 95°C for 10 min for hot-start polymerase activation, followed by 40 cycles of denaturation at 95°C for 15 s, annealing at 60°C for 1 min, and a melting curve stage as default setting from 60 to 95°C. Twelve samples were analyzed for each group, duplicate qPCR reactions were performed. The average CT value obtained from each primer pair was used to calculate the proportion of bacterial taxa over total bacteria in mouse feces. The data were transformed into a percentage using the ΔΔCT-based following formula as described elsewhere (Yang et al., 2015):

$$\begin{array}{c} X=\frac{{\left(Eff.Univ.\right)}^{C\,T\,u\,n\,i\,v}}{{\left(Eff.Spec.\right)}^{C\,T\,s\,p\,e\,c}}\times 100 \end{array}$$

Where *Eff. Univ.* is the calculated efficiency of the universal primers and *Eff. Spec.* refers to the efficiency of the taxon-specific primers. *CT univ* and *CT spec* are the CT mean values registered by the Fast real-time thermocycling platform. “X” represents the percentage of 16S taxon-specific copy number existing in each fecal sample. Finally, spearman correlations analyses were performed for comparing the data from 16S rRNA sequencing versus the 16S rRNA relative proportion obtained by qPCR; *p* values <0.05 were considered statistically significant.

### Taxonomic Differential Abundance Analysis

To detect differences in richness and α-diversity between groups, either Mann–Whitney *U* tests or Kruskal–Wallis test were performed among sample categories while measuring the observed estimates of α-diversity (richness of unique ASVs). Stratified permutational multivariate analysis of variance (PERMANOVA) with 999 permutations was conducted on all principal coordinates that were obtained during PCoA with the *Adonis* function of the *vegan* package in R, to observe the statistical significance (*p* < 0.05) of clusters according to the sample categories. To understand multivariate homogeneity of groups dispersion (variances) between multiple conditions, *Vegan’s betadisper* function was performed with pairwise comparisons of group mean dispersion.

Differential abundance analyses of taxa between cranberry and blueberry-fed mice and HFHS-fed group was determined at finer taxonomic level (the ASVs level) using the *DESeq2* package in R (Love et al., 2014). Only taxa found to be significant (*p* < 0.05 after multiple-hypothesis testing) were reported. *DESeq2* is a statistical method for differential analysis of count data, using shrinkage estimation for dispersion and fold changes while accounting for library size differences and biological variability (Love et al., 2014). It has recently been demonstrated that adopting these methods to microbiome taxonomy count data, as a direct analogy to differential expression from RNA-Seq, leads to improvements in detecting differential abundance compared to simple proportions or rarefying (McMurdie and Holmes, 2014). Results were expressed as log_2_-fold change in HFHS relative to Chow-fed mice and in CP, CF, BP, and BF groups relative to HFHS-fed mice. To detect differentially abundant features, relative abundance comparisons at the genus, family, and phylum levels were performed on normalized data, employing non-parametric Mann–Whitney *U*-tests, as well as, Kruskal–Wallis test with multiple comparison correction according to FDR method of Benjamini and Hochberg (Prism 8.0, GraphPad software, California). Similarly, those tests were used as appropriate to analyze the changes in the relative proportion of *A. muciniphila* examined by qPCR analysis in all the groups.

Comparisons at finer taxonomic levels (family through genus) were performed with linear discriminant analysis (LDAs) by LEfSe to identify microbial taxa biomarkers (Segata et al., 2011), that characterize the differences between groups of mice. The *p*-value for Kruskal–Wallis and Wilcoxon tests was set at 0.05, the logarithmic LDA score threshold was 2.0, and per-sample normalization of sum values was applied (LEfSe default parameters). These biomarkers are microbial taxa that differ in abundance between groups, as identified by a Wilcoxon rank-sum test. The effect size of each biomarker was then estimated by determining an LDA score (Segata et al., 2011). Alike, LEfSe analysis and *q*-value were employed to identify microbial functions and KEGG pathways statistically over-represented in cranberry and blueberry-fed mice compared to HFHS-fed mice.

### Mouse Phenotypes Statistical Analysis

To compute individual pairwise comparisons of means of histological analysis, Student’s *t*-tests and one-way ANOVA with a Dunnett *post hoc* test (Graph Pad Prism 8.0) were performed between the berries-enriched diets CP, BP and the fiber-enriched diets CF and BF compared to HFHS control group. Significance changes on the metabolic phenotypes (OGTT, ITT) between groups at different time points were calculated using a two-way repeated measures ANOVA with Dunnett’s *post hoc* test correction (Graph Pad Prism 8.0). *p*-values less than 0.05 were considered statistically significant whereas *p*-values between 0.05 and 0.1 were considered as showing a trend. Results were expressed as means ± standard error of the mean (SEM).

## Results

### Phytochemical Composition of the Whole Cranberry and Blueberry Powders

The complete phenolic characterization of berry powders and their fibrous fractions are presented in the Supplementary Tables S1, S2. In order to compare the prebiotic effects of fibers with polyphenol-rich berry powders, the quantity (g/100 g) of each fibrous fraction added to a HFHS-diet were balanced to provide an equal amount of fibers as encountered in their respective berry powder. Diets were adjusted to be isocaloric with a total energetic density of 5.4 kcal/g (Table 1).

### Polyphenol-Rich CP Reduced Body Weight, Fat Mass and Energy Efficiency While CF Decreased Liver Triglycerides in HFHS-Fed Mice

The HFHS-diet consumption induced a significant increase of mice BW from the week 5 until the end of the study as compared with those of the Chow diet-fed counterparts (Supplementary Figures S1A,B). Importantly, polyphenol-rich CP reduced the total BW gain after 8-weeks (*p* < 0.05 vs HFHS) and fiber-rich CF tended to reduce it (*p* = 0.0826 vs HFHS). A significant decrease in body fat mass (percentage of BW) and energy efficiency (ratio of BW to energy intake) was consistent with a lower BW in CP-fed mice (*p* < 0.01 vs HFHS) (Supplementary Figure S1). CP lowered the IWAT weights relative to final BW (*p* < 0.01 vs HFHS) and trended to reduce the EWAT (*p* = 0.0847 vs HFHS) (Supplementary Figures S1E,F). Moreover, no significant changes in BW of BP and BF fed mice were observed as compared to HFHS. As a matter of fact, an increase of EWAT in BF-fed mice was observed (*p* < 0.05 vs HFHS) (Supplementary Figure S1F). Furthermore, HFHS-diet significantly increased mice TG levels (*p* < 0.001 vs Chow). Interestingly, fiber-rich CF-fed mice had lower liver TG compared to HFHS (*p* < 0.05). However, an increased TG was observed in fiber-rich BF-fed mice (*p* < 0.05 vs HFHS) (Supplementary Figure S1I).

### Polyphenol-Rich CP and BP and Their Fibrous Fractions did Not Affect Glucose Metabolism Disorder in HFHS-Fed Mice

After a glucose load, glycemia level reached 25 mmol/L after 15 min and remained significantly higher during the entire OGTT in the mice fed HFHS-diet as compared to Chow-fed mice (*p* < 0.001) (Supplementary Figures S1J–K). However, CP, BP, CF, and BF did not affect glycemia in obese mice, showing similar AUC relative to HFHS (Supplementary Figure S1K). Plasma insulin during the OGTT were increased in HFHS (*p* < 0.05 vs Chow) (Supplementary Figures S1L,M). Likewise, fasting glycemia and insulinemia were higher in HFHS than in Chow fed mice (*p* < 0.05, Supplementary Figures S1N,O). Only BF-fed mice presented increased fasting insulin as compared to HFHS.

Insulin tolerance test was performed in the same groups to assess the effects of berry constituents on HFHS diet-induced insulin resistance. HFHS-fed mice had a decreased ITT at 5 and 10 mins timepoints of the ITT compared to Chow (*p* < 0.05) (Supplementary Figure S1P), but the AUC indicated that both HFHS and Chow similarly responded with lower glycemia during the 60 min after insulin challenge (Supplementary Figure S1Q). Likewise, CP and BP fed mice, as well as CF and BF fed mice, had similar response to exogenous insulin, with no significant changes compared to HFHS (Supplementary Figure S1Q). Insulin resistance, as reflected by the homeostasis model assessment of insulin resistance (HOMA-IR) index, was higher in HFHS-fed mice compared to Chow (*p* < 0.05). Polyphenol-rich CP, BP and their fiber-rich CF and BF did not significantly affect this index compared to HFHS (Supplementary Figure S1R).

### Polyphenol-Rich BP Improved Colonic Histomorphology in HFHS-Fed Mice

Representative histological sections of colon tissues of treated mice are presented in Supplementary Figures S2A–F. HFHS-diet had a detrimental effect on the colonic mucus thickness as compared to Chow (*p* < 0.01). Here, BP improved mice mucus thickness (*p* < 0.01 vs HFHS). However, there were no differences in the crypt depth and number of GC per crypt depth (μm^2^) among CP, BP, CF, and BF fed mice as compared to HFHS and Chow fed mice (Supplementary Figures S2H,I). Since both the commensal bacteria and diet influence host-derived mucin (Schroeder, 2019), we evaluated the proportion of acid, neutral and mixed mucins. The GC stained either by AB or periodic-acid Schiff (PAS), specific for sulfated and sialylated type of acidic mucins (blue), and α-glycol neutral mucins (magenta), respectively (Deplancke and Gaskins, 2001), were evaluated; GC stained with purple color indicated the presence of both neutral and acid mucins. HFHS-fed mice presented an increased proportion of mixed mucin (neutral and acid mucins) (*p* < 0.05 vs Chow) (Supplementary Figure S2J). HFHS-diet tended to decrease neutral mucin-filled GC (*p* = 0.0916 vs Chow). However, there was no significant difference in the types of mucin-filled GC between mice fed CP, BP, CF and BF compared to HFHS.

Positive effects of berry constituents on the cecum content weight was measured as indicative of dietary fibers fermentation and gut microbiota changes (Adam et al., 2014). As expected, HFHS-diet lowered mice cecum weight (*p* < 0.0001 vs Chow) (Supplementary Figure S2K). Both berry fiber-rich fractions did not alter the cecum content compared to HFHS. Polyphenol-rich CP increased the cecum mass in obese mice (*p* < 0.001 vs HFHS). Even though polyphenol-rich BP tended to increase the cecum mass, it was not statistically significant (162.2 ± 5.7 mg, *n* = 12 vs 140 ± 140.4 in HFHS) (Supplementary Figure S2K).

### Polyphenol-Rich CP and BP and Their Fibrous Fractions CF and BF Increased Gut Microbial Richness and Diversity in HFHS-Fed Mice

**FIGURE 1:**
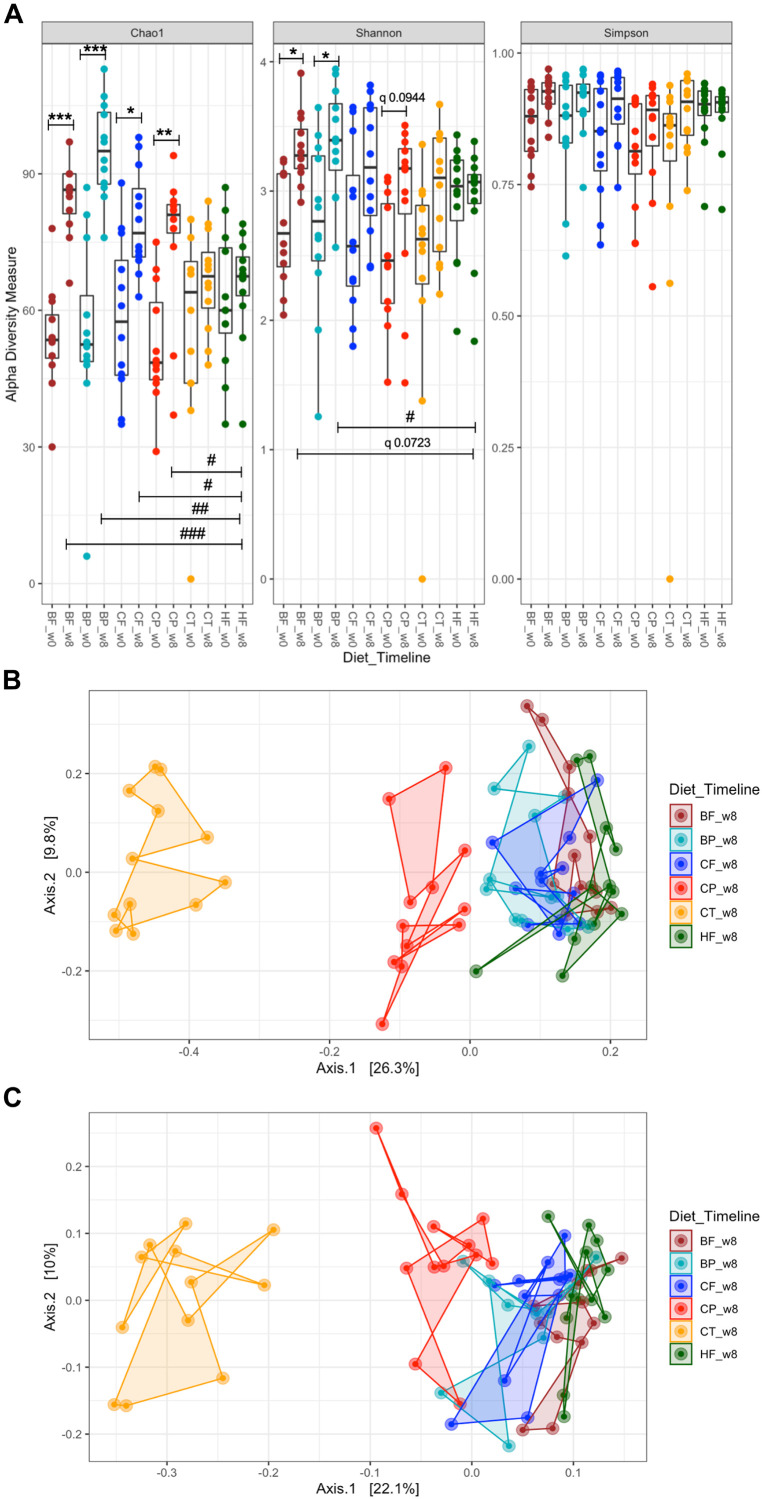
Polyphenol-rich CP and BP and their fiber-rich CF and BF distinctly influenced the gut microbiota structure of HFHS-induced obese mice. Mice were fed a HFHS-diet (HF), Chow (CT), polyphenol-rich whole cranberry powder (CP), cranberry fiber-rich fraction (CF), polyphenol-rich whole blueberry powder (BP) and blueberry fiber-rich fraction (BF). **(A)** α-diversity, determined by Chao1, Shannon-diversity and Simpson-evenness plotted for each dietary group at 0 and at 8-weeks. Line inside the box represents the median, while whiskers represent the lowest and highest values within 1.5 interquartile range (IQR); **(B)** Principal Coordinates Analysis (PCoA) plot of Bray–Curtis dissimilarities between samples at 8-week (PERMANOVA *R*^2^ 0.40295, *p* < 0.001); **(C)** PCoA of taxon phylogenetic tree-based unweighted UniFrac of samples at 8-week (PERMANOVA *R*^2^ 0.3354, *p* < 0.001). α-diversity significances are represented as *p < 0.05, **p < 0.01, and ***p < 0.001 as compared to baseline within the same group, and ^#^*p* < 0.05, ^##^*p* < 0.01, and ^###^*p* < 0.001 as compared to HFHS at 8-week. Corrected *p*-values having a tendency are indicated as “*q*”. Each sample point (*n* = 12 per group) is color-coded based on the administered diet for 8-weeks, as shown in each figure legend.

Changes in α-diversity of mice gut microbiota were contrasted against both the baseline (0-week) and with HFHS-fed mice at 8-week. HFHS did not significantly affect microbial richness (*Chao1*) and *Shannon* diversity as compared to baseline (Figure 1). Importantly, CP and BP, as well as CF and BF, increased microbial richness relative to both the baseline and to HFHS-fed mice (*p* < 0.05) (Figure 1A). BF increased microbial diversity compared to baseline (*Shannon* index, *p* < 0.05); yet, it tended to increase this parameter when compared to HFHS at 8-week (*q* = 0.0723). Particularly, polyphenol-rich BP increased gut microbiota diversity after 8-week (*p* < 0.05 vs HFHS) (Figure 1A); at the same time, CP tended to increase it (*q* = 0.0944). All treatments slightly improved *Simpson* evenness compared to HFHS, yet not significantly.

### Polyphenol-Rich CP and BP Influenced Gut Microbiota Structure in HFHS-Fed Mice

Bray–Curtis β-diversity metrics among mice gut microbiota at 8-week, revealed different hierarchical clustering in the PCoA plot (Figure 1B). Particularly, CP distinctly drove the gut microbiota structure away from HFHS-fed mice, including when the taxon-based UniFrac measure was integrated into the analyses (Figure 1C). In a similar manner, gut microbiota of BP-fed mice diverged from that of HFHS (Figure 3C). CF and BF diets only slightly affected the gut microbiota structure as compared to HFHS (Figures 1B,C). PERMANOVA revealed a *R*^2^ of 0.4030 (*p* < 0.001) for Bray–Curtis dissimilarity and *R*^2^ 0.3354 (*p* < 0.001) for UniFrac phylogenetic-based metrics.

### Polyphenol-Rich CP and BP and Not Their Fibrous Fractions Decreased *Firmicutes* and Prompted *Verrucomicrobia* and *Actinobacteria* Phyla in HFHS Fed Mice

At the phylum level, HFHS induced a higher proportion of *Firmicutes* (*q* < 0.0001, 76.2 ± 3.5% vs 60.9 ± 4.1% Chow) and lower the proportion of *Bacteroidetes* (*q* < 0.0001, 6.7 ± 2.0% vs 29.3 ± 3.2% Chow) (Supplementary Figure S3A). Remarkably, both CP and BP decreased *Firmicutes* (*q* < 0.0001, 55.6 ± 4.9% and 59.6 ± 4.4%, respectively) and prompted *Verrucomicrobia*, nearly doubling the proportion to 28.1 ± 3.9% in CP and 25.4 ± 3.6% in BP fed mice as compared to 14.9 ± 2.7% in HFHS (*q* < 0.05). A significant increase of *Actinobacteria* was also observed in mice fed polyphenol-rich CP (*q* < 0.05, 11.6 ± 2.0%), likewise, BP tended to favor this phylum (9.3 ± 1.9%, *q* = 0.0533 vs 4.5 ± 0.8% HFHS) (Supplementary Figure S3A).

### Polyphenol-Rich CP and BP Triggered Health-Promoting Taxa in HFHS-Fed Mice

Pairwise comparisons (Kruskal–Wallis test with *pos-hoc* corrections) at the family and genus level were performed between groups at 8-week. Summary bar plots (Figure 3B) indicate the proportion of bacteria families and genera per dietary group. In Chow-fed mice, *Muribaculaceae* (29.3 ± 3.2%) and *Lactobacillaceae* (17.0 ± 4.7%) covered the majority of gut microbes. However, HFHS dropped these families (*q* < 0.0001, *Muribaculaceae* 6.7 ± 2.0% and *Lactobacillaceae q* < 0.05, 7.7 ± 4.7%). On the contrary, HFHS-fed mice had higher proportion of *Lachnospiraceae* (*q* < 0.0001, 26.0 ± 2.5% vs 7.0 ± 1.0% Chow), *Ruminococcaceae* (*q* < 0.01, 21.0 ± 3.8% vs 9.0 ± 1.3% Chow), and *Peptostreptococcaceae* (*q* < 0.05, 5.8 ± 2.4% vs 0.1 ± 0.1% Chow). Surprisingly, when compared to HFHS, CP inhibited the growth of *Lachnospiraceae* (*q* < 0.0001, 8.7 ± 1.3%), *Ruminococcaceae* (*q* < 0.05, 11.3 ± 1.7%) and *Peptostreptococcaceae* (*q* < 0.05, 0%), keeping their relative proportion close to those observed in Chow (Supplementary Figure S3B); this was not the case for CF-fed mice. Although BP also contained polyphenols, this diet did not lower the abundance of above HFHS-induced pathobionts. Alike, there were no differences of other taxa at the family level in fiber-rich BF-fed mice as compared to HFHS.

Polyphenol-rich CP and BP significantly promoted taxa known to confer beneficial effects to host health. Selective prebiotic-like effects on *Akkermansiaceae* and *Coriobacteriales_Incertae_Sedis* were favored by CP and BP, but this outcome was not triggered by their fibrous fractions. At 8-week, *Akkermansiaceae* was by 10.0 ± 4.3% in Chow and 12.6 ± 1.6% in HFHS fed mice. Remarkably, *Akkermansiaceae* was increased in CP and BP-fed mice in 28.1 ± 3.9% and 25.4 ± 3.6%, respectively (*q* < 0.05 vs HFHS); on the opposite, mice fed fiber-rich CF and BF had no significant changes (16.2 ± 3.9% and 15.1 ± 2.8%). While the polyphenol-degrading taxa *Coriobacteriales_Incertae_Sedis* was undetected in both Chow and HFHS fed mice at 8-week, it was distinctly promoted by CP up to 9.1 ± 1.9% and BP up to 5.2 ± 1.5% in HFHS-fed mice (*q* < 0.000). Importantly, CF also stimulated *Coriobacteriales* (*q* < 0.05), indicating that residual non-extractable polyphenols were present in cranberry fibers, likely, forming non-covalent interactions with cell wall polysaccharides. This finding was further confirmed by analyzing PACs content in blueberry and cranberry fibrous fractions, showing the presence of non-extractable PACs in CF (Supplementary Table S3).

### Polyphenol-Rich Diets Exert Selective Prebiotic Effects on *A. muciniphila*, *D. newyorkensis*, and *Coriobacteriales_Incertae_Sedis* in HFHS-Fed Mice

By analyzing ASVs at a finer-taxonomic level, 59 genera were identified in mice gut microbiota (Supplementary Figure S3C). Within the taxa distinctly modulated, *A. muciniphila* was selectively prompted in polyphenol-rich CP and BP fed mice (*q* < 0.05 vs HFHS). These results were confirmed by qPCR-amplifying *A. muciniphila* 16S rRNA gene copy, which relative proportions were significantly correlated with 16S rRNA sequencing data (spearman *R*^2^ > 0.902, *p* < 0.0001, Supplementary Figures S3D,E). *Lactobacillus* (12.4 ± 5.2%), *Coriobacteriales_Incertae_Sedis* (9.1 ± 1.9%), *Lachnospiraceae_NK4A136_group* (9.0 ± 1.4%), and *Dubosiella* (3.5 ± 1.1%) were particularly stimulated in CP-fed mice. In BP-fed mice, *Lachnospiraceae_NK4A136_group* (4.8 ± 0.9%) and *Coriobacteriales_Incertae_Sedis* (5.2 ± 1.5%) were predominant.

**FIGURE 2:**
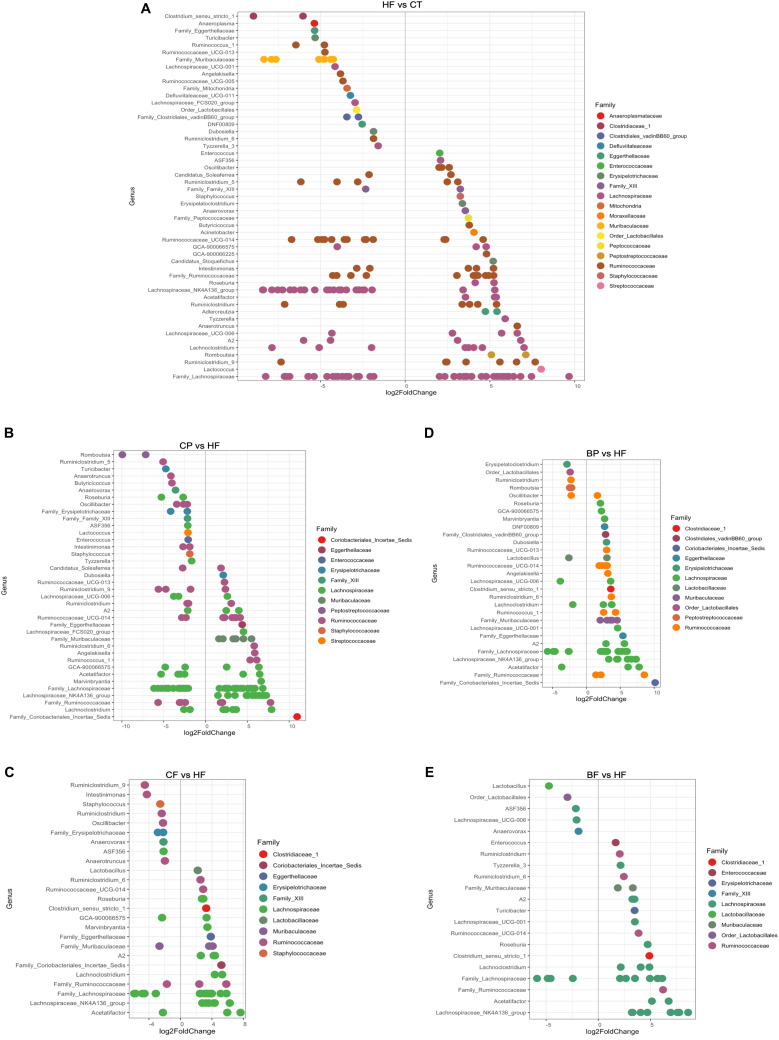
Taxa significantly up-regulated and down-regulated by polyphenol-rich CP and BP and their fiber-rich CF and BF. **(A)** Differential abundance analysis (DESeq2) identified 51 ASVs significantly modulated in mice fed HFHS (HF) relative to lean mice fed Chow (CT); **(B)** 38 ASVs were modulated in mice fed polyphenol-rich whole cranberry powder (CP) relative to HFHS; **(C)** 25 ASVs were modulated in mice fed cranberry fibe-rich fraction (CF) relative to HFHS; **(D)** 29 ASVs were modulated in mice fed polyphenol-rich whole blueberry powder (BP) relative to HFHS; **(E)** 21 ASVs were modulated in mice fed blueberry fiber-rich fraction (BF) relative to HFHS. ASVs (*p* < 0.05, FDR-corrected) are represented by single data points (with some data points overlapping) within each genus grouped on the *x*-axis, and by color fitting to which taxonomic family the ASVs belong. Data are plotted as log2 fold change (*n* = 12 mice per group). ASVs to the right of the zero line are more abundant and ASVs to the left of the zero line are less abundant.

Considering that a large number of genera were modulated in mice microbiota, *DESeq2* differential analysis was used to estimate the taxa fold changes after 8-week intervention, while accounting for library size and biological variability (Love et al., 2014) of berry-based diets compared to HFHS. Only genera and related families significantly changed by more than twofold after *p*-values adjustment (FDR Bonferroni correction, *q* < 0.05) are presented in Figure 2. HFHS differentially influenced 51 genera as compared to Chow (Figure 2). CP repressed the HFHS-induced pathobionts of *Romboutsia*, *Ruminiclostridium*, *Roseburia*, *Oscillibacter*, *Lactococcus*, and *Butyricicoccus*. Interestingly, CP increased by 5- and 10-fold polyphenol-degrading taxa *Eggerthellaceae* and *Coriobacteriales_Incertae_Sedis* respectively, as compared to HFHS (Figure 2B). Likewise, there were 2- up to 8-fold higher abundance of *Angelakisella*, *Muribaculaceae*, *Dubosiella*, and *Lachnospiraceae_NK4A136_group* in CP-fed mice. Similarly, BP inhibited *Romboutsia*, *Ruminiclostridium*, and *Oscillibacter* compared to HFHS, and prompted both a 5- and a 10-fold increase of *Eggerthellaceae* and *Coriobacteriales_Incertae_Sedis* (Figure 2D).

The most promoted taxa by CF and BF were *Lachnospiraceae_NK4A136_group* and *Acetatifactor*, being 7- and 8-fold higher than in HFHS, respectively. Considering their high content in fibers, these diets promoted polysaccharide-degrading taxa, such as *Clostridium_senso_stricto_1*, *Muribaculaceae*, and *Roseburia* (Figure 2C). Fiber-rich CF inhibited *Ruminiclostridium*, *Oscillibacter*, and *Anaerotruncus* pathobionts while BF dropped *Anaerovax* and *Lactobacillus*, as compared to HFHS (Figure 2E). However, in contrast to CF, the BF did not favor polyphenol-degrading taxa *Eggerthellaceae* and *Coriobacteriales_Incertae_Sedis*, as found on both cranberry constituents CP and CF.

**FIGURE 3:**
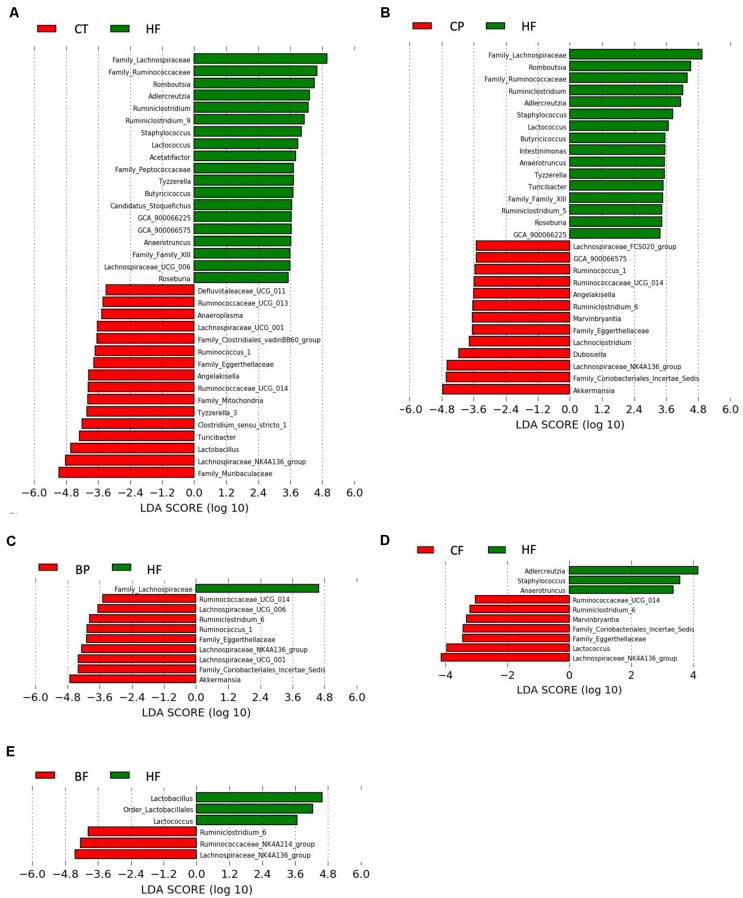
Bacterial biomarkers in mice fed the polyphenol-rich CP and BP and their fiber-rich CF and BF. Key phylotypes were identified using linear discriminant analysis (LDA) combined with effect size (LEfSe) algorithm. **(A)** LEfSe analysis showing taxa distinguishing Chow-fed mice (CT) as compared to HFHS (HF); **(B)** Taxa distinguishing mice fed polyphenol-rich whole cranberry powder (CP); **(C)** Taxa distinguishing mice fed polyphenol-rich whole blueberry powder (BP); **(D)** Taxa distinguishing mice fed fiber-rich cranberry fraction (CF); and **(E)** Taxa distinguishing mice fed fiber-rich blueberry fractions (BF). Threshold for LDA score was 2.0. Positive LDA (green bars) are enriched in HF, while negative LDA (red bars) are enriched in CT, CP, BP, CF, and BF as shown on figure legends (*n* = 12 mice per group). Statistically significant taxa enrichment was obtained with Kruskal–Wallis test among classes (*p*-value = 0.05).

Using LEfSe analysis (Figure 3), *A. muciniphila* was revealed as main biomarker discriminating the gut microbiota composition of polyphenol-rich CP and BP fed mice. In addition, *Coriobacteriales_Incertae_Sedis, Lachnospiraceae_NK4A136_group*, *Eggerthellaceae*, and *Ruminococcaceae_UCG_014* were characteristics in CP and BP fed mice. Noteworthy, CP specifically prompted *Angelakisella*, and *Dubosiella* genera. *Lachnospiraceae_NK4A136_group* was the most representative taxon in both CF and BF. It is worth noting that within the fiber-rich diets, only CF revealed as biomarkers the polyphenol-degrading *Coriobacteriales_Incertae_Sedis* and *Eggerthellaceae*.

### The Type of Diet-Enrichment and Signature Taxa Discriminate the Enterotype-Like Clustering and Body Weight Changes in Mice

**FIGURE 4:**
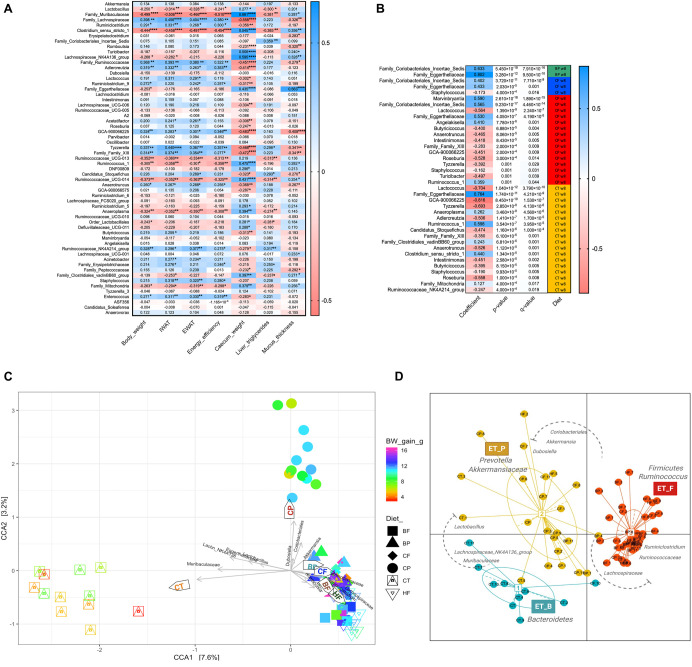
Widespread prebiotic role of polyphenols and fibers on bacterial taxa and their correlations with mice phenotypes and microbiota enterotype-like clustering. **(A)** Heatmap showing microbial associations (spearman *R*^2^ scores) with mice obesity phenotypes. Each row represents the effects of one of the 59 ASVs (>0.05% relative abundance) in each of the mouse phenotypes in columns. Asterisks indicate taxa for which the association was significant, *p < 0.05, **p < 0.01, ***p < 0.001, and ****p < 0.0001. **(B)** Taxa showing opposing or compatible connection with the type of diet supplementation based on MaAslin model applied for uncovering the prebiotic effects of berry constituents. Rows represent different taxa exhibiting main interaction with each of the type of diet supplementation. Columns represent coefficient scores, *p*-value (*p* < 0.05), FDR corrected *p*-value (*q* < 0.05) and diet interaction. Taxa showing compatible correlation are indicated in blue color and those negatively correlated are indicated in red color. **(C)** Ordination of Canonical Correspondence Analysis (CCA) showing the connection between samples and environmental variables along with representative taxa significantly modulated by the diets in mice gut microbiota. Mice were fed a HFHS-diet (HF), Chow (CT), polyphenol-rich whole cranberry powder (CP), cranberry fiber-rich fraction (CF), polyphenol-rich whole blueberry powder (BP) and blueberry fiber-rich fraction (BF). Arrows indicate environmental variables and taxa discriminating the dietary groups: Mucus_thickness, IWAT, Liver_TG, Energy_efficiency, *Lactobacillus*, *Muribaculaceae*, *Lachnospiraceae*, *Lachnospiraceae_NK4A136*, *Eggerthellaceae*, *Coriobacteriales*, *Dubosiella*, and *Akkermansia*. Symbol colors of each group represents changes in mouse body weight gain (BW). **(D)** Ordination on Jensen-Shannon metrics shows enterotype-like clustering of mice gut microbiota (colored ellipses); dots represent abundance distributions of bacteria genera from each mouse. Three enterotypes were identified: (1) ET_B, *Bacteroidetes/Muribaculaceae*; (2) ET_P, *Prevotella/Akkermansiaceae*; and (3) ET_F, *Firmicutes/Ruminococcus*. Taxonomic drivers of enterotypes are indicated in the plot (see Supplementary Figure S4).

To identify specific taxa associated with mice phenotypes and diet-supplementation, spearman rank correlations, CCA and enterotype-like functional clustering (Costea et al., 2018) were performed (Figure 4). Bacterial taxa of *Muribaculaceae*, *Lactobacillus*, *Lachnospinaceae_NK4A136_*group and *Clostridium_sensu_stricto_1*, negatively correlated with BW gain, adipose tissue accretion, energy efficiency and liver TG (*p* < 0.05). Interestingly, among polyphenol-degrading families, *Eggerthellaceae* was inversely correlated with BW gain (*p* < 0.05) and positively correlated with an improved cecum size and mucus thickness (*R*^2^ = 0.435 and 0.563, respectively, *p* < 0.0001). On the opposite, *Lachnospinaceae*, *Ruminococcaceae*, and *Ruminiclostridium* were positively correlated with BW gain, energy efficiency and reduced cecum size, which is consistent with HFHS-induced obesity, characterized by an increased proportion of these taxa.

Confirming the causal effect of diet on the gut microbiota composition and its link with changes in mice phenotypes, the CCA diagram and enterotype-like clustering reveal divergent microbial key players in Chow and CP fed mice from those observed in HFHS (Figures 4C,D). Surprisingly, *Muribaculaceae*, *Lachnospiraceae_NK4A136_group*, *Egger-thellaceae*, and *Lactobacillus* assembled with Chow-group and correlated with mucus thickness. Likewise, *Dubosiella*, *Coriobacteriales_Incertae_Sedis*, and *Akkermansia* correlated with CP and differed from HFHS within the CCA. In this sense, HFHS, as well as BP, BF and CF clustered in the same direction of the BW gain, the accretion of adipose tissues (IWAT and EWAT) and energy efficiency increase; *Lachnospiraceae* characterized this last cluster. PERMANOVA *R*^2^ score was 0.36472 (*p* < 0.0001). The enterotype-like clustering revealed the *Firmicutes/Ruminococcus* enterotype (ET-F) linked to HFHS-fed mice gut microbiota; *Lachnospiraceae* (25 ± 1.3%) and *Ruminococcaceae* (8 ± 0.82%) were the main taxa characterizing ET_F (Figure 4D and Supplementary Figure S4). Importantly, HFHS-diet enrichment with CP shifted the mice gut microbiota into *Prevotella/Akkermansiaceae* enterotype (ET_P), which was shared with mice fed Chow-diet. Interestingly, *Akkermansia* (25 ± 3%) and *Lactobacillus* (19 ± 4.5%) were the main taxonomic drivers of ET_P, while *Muribaculaceae* (34 ± 2.6%) and *Lachnospiraceae_NK4A136_group* (16 ± 2.3%) were the signature taxa of *Bacteroidetes/Muribaculaceae* enterotype (ET_B), mainly encompassing the lean mice gut microbiota.

Using a multivariate association with linear models (MaAsLin) (Mallick et al., 2019), changes in taxa proportion were tested for their dependence on polyphenol-rich and fiber-rich diets, demonstrating the main effects of the type of berry constituents on promoting specific taxon (Figure 4D). Interestingly, *Coriobacteriales_Incertae_Sedis* and *Eggerthellaceae* stood out as representative of BP and CP, underlining a significant species/polyphenol interaction (*q* < 0.0001). Moreover, *Eggerthellaceae* was strongly correlated with Chow-diet (*R*^2^ = 0.764, *q* < 0.0001). Most of taxa increased by HFHS, were negatively correlated with Chow and CP diets.

### Polyphenol-Rich CP Diet Is Associated With Predicted Functions of Gut Microbiota in HFHS-Fed Mice

**FIGURE 5:**
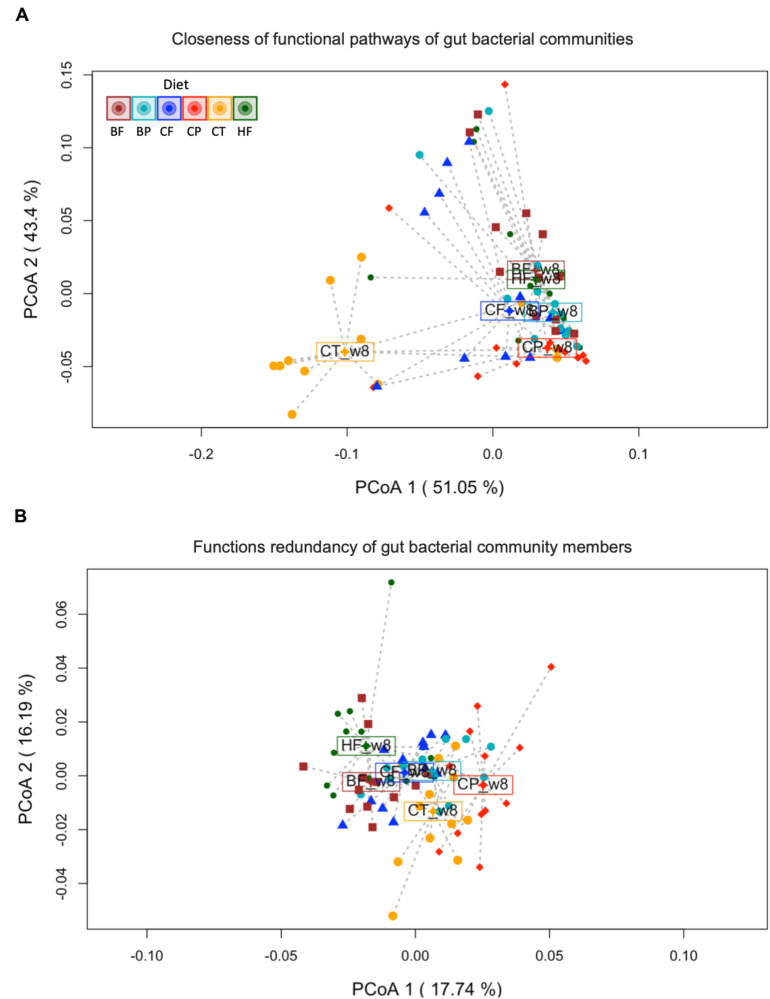
Gut microbiota structure of mice fed polyphenol-rich CP is functionally distinct from HFHS-induced obese mice. **(A)** Principal Coordinates Analysis (PCoA) on Bray–Curtis metrics indicating the closeness of predicted functions of gut bacterial communities. PERMANOVA *R*^2^ 0.30065, ***p < 0.001. **(B)** PCoA on Bray–Curtis metrics of functional redundancy of gut bacterial members according to the type of diet supplementation. Constrained distance-based redundancy shows a clear separation of the functional profiles of mice fed polyphenol-rich whole cranberry powder (CP) and Chow (CT) from HFHS (HF) group. Mice fed the polyphenol-rich whole blueberry powder (BP), blueberry fiber-rich fraction (BF) and cranberry fiber-rich fraction (CF) were confined together slightly separated from HF. PERMANOVA *R*^2^ 0.27394, ***p < 0.001. Dots represent microbiome samples, colored by diet (*n* = 12 mice per group). Each centroid (enclosed diet name) is representing the mean functional/redundancy composition of each group.

Variations in gut microbiota functions of mice fed polyphenol-rich CP and BP and their fiber-rich CF and BF were evaluated based on Bray–Curtis dissimilarities and LEfSe analysis of functional profiles obtained by Tax4Fun2 (Wemheuer et al., 2018). At 8-week, gut microbiota KEGG functions (levels 1 and 2) of HFHS-fed mice differed from those fed Chow-diet, irrespective of the choice of analytical statistical method (LEfSe and PERMANOVA) (Supplementary Figures S5–S7 and Figure 5A). Surprisingly, CP-fed mice clearly shifted away from HFHS-fed group and remained positioned at the level of Chow-fed mice within the PCoA (axis 2, 43.4% variation), indicating that the CP mice gut microbiota had a functional structure comparable to that of the Chow-group (Figure 5A). Moreover, BP and fiber-rich CF fed mice had similar function patterns, as indicated by the proximity of their centroids; however, they slightly moved away from HFHS. Conversely, fiber-rich BF did not impact gut microbial functions of HFHS-fed mice, remaining closely linked to HFHS centroid in the PCoA. PERMANOVA for functional β-diversity showed a *R*^2^ of 0.3007 (*p* < 0.001). The *betadisper* results showed no significant differences in the dispersion of mice within each group (*permutest F* value 0.8317, *p* = 0.514), demonstrating that the PERMANOVA-obtained *p*-value was strongly influenced by changes in the taxonomic functional structures, and was not an artifact of heterogeneous dispersion of group’s variances.

### CP-Diet Drive Distinct Metabolic Functions by Coexisting Bacterial Taxa in HFHS-Fed Mice

We further analyzed the functional redundancy (FRI) of gut microbiota to evaluate the coexistence of taxonomic assigned-sequences capable of fulfilling analogous metabolic functions. The FRI was obtained by Tax4fun2 analysis and results were plotted in a PCoA based on Bray–Curtis metrics (Figure 5B). In particular, CP cluster markedly diverged away from the HFHS in the PCoA, suggesting that metabolic functions were distinctively represented by taxa prompted by CP-diet. FRI showed a less pronounced separation amongst BP, BF and CF centroids and that of the HFHS. This demonstrates that predicted metabolic functions are strongly conserved in the bacterial communities identified in all aforementioned groups, although the relative abundance and gut taxonomic composition differed across BP, BF and CF diets (as shown by β-diversity and *DESeq2* analysis; Figures 1, 2). PERMANOVA based on FRI was *R*^2^ 0.2740 (*p* < 0.001). Likewise, homogeneity of dispersion within groups was confirmed (*permutest F* = 0.6323, *p* = 0.679).

LEfSe analysis (Supplementary Figures S6, S7) based on the KEGG level 2, showed 10 functional pathways significantly overrepresented in the gut microbiota of HFHS-fed mice. Genes encoding for energy metabolism (mainly sulfur metabolism, nitrogen metabolism and methane metabolism at the KEGG level 1), pathways of global and overview maps, and amino acid metabolism were featured in HFHS-fed mice as compared to Chow (Supplementary Figures S5A, S6). In contrast, CP favored functions involved in antimicrobial drug resistance, and membrane transport, among others as compared to HFHS (Supplementary Figures S5B, S7A). In case of BP, pathways of metabolism of cofactors and vitamins, lipid metabolism and DNA replication and repair were significantly enriched when compared to HFHS (Supplementary Figure S5C). CF promoted nucleotide metabolism, amino acids metabolism and carbohydrate metabolism among others functional pathways. However, BF only revealed two pathways being significantly modulated as compared to HFHS (Supplementary Figures S5D,E).

### Microbial Functions Profile Correlates With Improved Liver TG, Energetic Efficiency and Body Weight Changes

We evaluated the link between mice phenotypes (liver TG, energetic efficiency, and BW gain) and microbial functional pathways via MaAsLin model. A number of enriched pathways was found negatively correlated with both BW gain and energy efficiency in mice (Supplementary Tables S5, S6). Relative abundances of 37 functional pathways (level 1) were correlated with BW changes (FDR *q* < 0.001). Within the top-3 functions, genes coding for amino sugar and nucleotide sugar metabolism, fructose and mannose metabolism, and glutathione metabolism were negatively correlated with BW gain. In contrast, pathways such as biosynthesis of secondary metabolites, biosynthesis of antibiotics, and biosynthesis of phenylalanine, tyrosine and tryptophan were positively correlated with BW gain. LEfSe further demonstrated significant over-representation of 48 functional pathways in HFHS-fed mice as compared to Chow (Supplementary Figure S6). These observations provide an explanation of how gut microbiota functions can attenuate obesity-associated phenotypes in HFHS-fed mice.

Liver TG was inversely correlated with the microbial pathways of degradation of aromatic compounds, ethylbenzene degradation, and alpha-linolenic acid metabolism (Supplementary Table S5). Genes coding for ABC transporters, starch and sucrose metabolism, and glycolysis/gluconeogenesis were negatively correlated to the energy efficiency.

## Discussion

The present study was helpful to appraise the prebiotic potential and influence of polyphenol-rich cranberry and blueberry powders from their fibrous fractions on obesity-associated disorders. We demonstrated that the polyphenol-rich CP reduced the adipose tissues, BW, and improved energy balance, without affecting food intake in HFHS-fed mice. However, these results were not reproduced by the fibrous fraction from the same fruit, indicating that cranberry polyphenols are largely contributing to counteract obesity phenotypes. In contrast, the BP diet did not induce significant changes on fat mass depots and BW, even if the polyphenol-rich CP and BP were incorporated to the HFHS-diet providing an equal caloric charge and total polyphenol content. In our study, CP was a mix of anthocyanins, phenolic acids, oligomers and PACs polymers (polymerization degree, DP 1–5 and >10, respectively). Particularly, PACs, which were the most abundant flavonoids in the cranberry powder used herein, exert strong effects on energy expenditure by acting on adipose tissues, skeletal muscle and liver (Caimari et al., 2013; Zhou et al., 2019). Here, CP suppressed HFHS-induced fat depots in IWAT, and to a less extent, in EWAT tissues. In support of our results, previous studies showed that cranberry polyphenols improved glucose homeostasis, lowered BW and liver TG in obese mice, and that such effects, were linked to the gut microbiota composition (Anhê et al., 2017).

High fat high sucrose-diet reduced the colonic mucus thickness and affected gut microbial richness and diversity. Importantly, BP improved mucus thickness, and all the berry-based diets increased microbial richness in obese mice. Our findings agree with several studies showing the influence of the gut microbiota in improving energy balance, metabolic function, intestinal barrier and BW changes in metabolic diseases (Masumoto et al., 2016; Zhao et al., 2017; Rodríguez-Daza et al., 2020).

When analyzing the gut microbiota at a deeper taxonomic level, individual taxa were modulated selectively in mice fed polyphenol-rich diets, but they were not modulated in mice fed fiber-rich diets. Particularly, *Peptostreptococcaceae* (*Romboutsia* genus), *Lachnospiraceae*, and *Ruminococcaceae* (including *Ruminiclostridium*, *Oscillibacter*, and *Butyricicoccus*), shown to dominate the gut microbiota after a high-fat diet intake, were associated to increasing mice BW (Kameyama and Itoh, 2014; Zhao et al., 2017; Qin et al., 2018). These commensal bacteria, able to induce intestinal inflammation or pathology in diet-induced obesity, diabetes or any gut environmental alteration (Gurung et al., 2020), are actually pathobionts; here, they were impeded by CP and BP diets. The suppressive effect of polyphenols against pathobionts appears as one of the mechanisms involved in preventing obesity-related gut dysbiosis. Noteworthy, we also highlight the prebiotic effect of polyphenols on taxa linked to a lean phenotype and intestinal homeostasis. Indeed, CP and BP diets selectively prompted *A. muciniphila*, implicated in improving metabolic phenotypes in obesity and diabetes, regulating gut inflammation and host immune system (Everard et al., 2013; Schneeberger et al., 2015; Ansaldo et al., 2019). Furthermore, *Muribaculaceae*, characteristic of Chow-fed lean mice, was stimulated by CP and BP in HFHS-fed mice and was negatively correlated with BW gain. Consistent to our findings, *Muribaculaceae* appears to be involved in the prevention of obesity (Cao et al., 2019). *Muribaculaceae* species (*Bacteroidetes* phylum), are specialized in degrading dietary carbohydrates and considered biomarkers of a healthy mouse microbiome (Yang et al., 2019); this species is drastically inhibited by HFHS as shown in other reports (Cao et al., 2019; Hou et al., 2019) and as observed in the present study.

The MaAslin model was useful to demonstrate how specific taxa are driven by HFHS-enrichment either with berry fibers or polyphenols; such results were based on FDR corrected significance of multivariate associations. Essentially, *Coriobacteriaceae* and *Eggerthellaceae* were triggered by polyphenol-enriched diets, yet *Eggerthellaceae* was also associated to Chow-diet, likely due to the high content of cereal derived fibers in the latter (cellulose, hemicellulose, and lignin, Harlan Teklad global diets 2018). Interestingly, *Dubosiella* and *Angelakisella* were prompted by both CP and BP. Surprisingly, *D. newyorkensis* (*Erysipelotrichaceae* family), is used as a patented probiotic for modulating weight loss and preventing metabolism and immunity associated diseases, such as obesity, diabetes, metabolic syndrome, and abnormal lipid metabolism (Cox and Blaser, 2018). Additionally, *Angelakisella* may be a biomarker of Chow-fed lean mice microbiota; it is decreased by 4.0-fold in HFHS-fed mice. *Angelakisella* is a newly described species isolated from human ileum (Mailhe et al., 2017), for which functional benefits remain to be explored.

Polyphenol-degrading bacteria have been shown to potentiate bioactive phenolic metabolites (González-Sarrías et al., 2017). Of particular interest is *Eggerthellaceae*, including the *Eggerthella*, *Paraeggerthella*, *Gordonibacter*, and *Slackia* genera (Gupta et al., 2013; Cho et al., 2016), whose xenobiotic metabolism is linked to positive effects in lipid metabolism and liver detoxification in mice (Claus et al., 2011; Clavel et al., 2014; Cho et al., 2016). Interestingly, *Eggerthellaceae* was 5-fold higher in mice fed Chow, BP, CP and CF as compared to HFHS, and correlated to lower BW and restored intestinal histomorphology in obese mice.

Fiber-rich diets can promote SCFA-producing genera contributing to lipid metabolism and preventing metabolic abnormalities in mice (den Besten et al., 2015). Noteworthy, CF lowered liver TG in obese mice, but this was not the case for the fiber-rich BF. Non-extractable PACs linked to cranberry fibers could provide added effect on both gut microbiota composition and functions. Supporting this interpretation, fibers and polyphenols from peach by-products ameliorated insulin resistance and hepatic steatosis and lowered TG in high-fat high-fructose fed obese rats (Rodríguez-González et al., 2018). In the present study, synergistic effects between cranberry fibers and polyphenols are inhibiting obesity-related pathobionts. Fiber-rich CF inhibited *Ruminiclostridium*, *Oscillibacter*, and *Anaerotruncus* genera that were increased in HFHS-fed mice; in contrast, fiber-rich BF did not affect their proportions, and neither improve obesity phenotypes. In this context, a recent review summarized how unabsorbed polyphenols are associated to fibers by non-covalent binding (Jakobek and Matić, 2018). In particular, because of their structure and spatial configuration, A-type PACs found in cranberry have a higher affinity to pectin (Mamet et al., 2018), a naturally occurring polysaccharide found in berries. In addition, cranberry oligosaccharides exert antimicrobial and anti-adhesive effects against pathogens (Sun et al., 2015), as do polyphenols (Lacombe et al., 2013b; Lacombe and Wu, 2017). Here, we demonstrated the antimicrobial action of cranberry fibers as reported elsewhere (Hotchkiss et al., 2015; Sun et al., 2015), and the influence of repressed pathobionts on mice metabolic status and BW, as sustained herein by the multivariate correlation analysis and gut microbiota enterotypes.

Consistent to other studies (Prior et al., 2008; Mykkanen et al., 2014), berry powders and fibrous fractions failed to improve HFHS-induced glucose intolerance. Several factors, including the shorter duration of dietary intervention (8 weeks), and the feeding of berry fruit powders rather than concentrated polyphenols and purified fibers, likely led to these results. Nevertheless, 8-week dietary exposure was sufficient to significantly modulate mice gut microbiota. Improvements of metabolic, intestinal and BW phenotypes were related to a distinct gut microbiota structure between berry enriched and HFHS groups. Within this framework, functional structure analysis based in Bray–Curtis metrics demonstrated that CP-fed mice had a distinct gut microbiota metabolic profile compared to HFHS. Polyphenol-rich CP prevented microbial metabolic alterations in HFHS-fed mice, bringing them closer to that of Chow group. Noteworthy, establishing the link between a long-term diet intake and the functional stratification of gut microbes, enterotype-like clustering of mice gut microbiota demonstrates that polyphenol-rich CP drive the obesity-associated microbiota from *Firmicutes-Ruminococcus* enterotype, into that of *Prevotella/Akkermansiaceae*, linked to a healthier host status (de Moraes et al., 2017). A recent study demonstrated that within the identified *Prevotella*-driven bacterial indicators, *A. muciniphila* has been found positively correlated to a lower plasma glucose (de Moraes et al., 2017). Interestingly, the *Prevotella* enterotype has been previously shown to concomitantly degrade gut mucin oligosaccharide (Wright et al., 2000). Likewise, the mucin-degrading *A. muciniphila*, the main taxonomic contributor of the ET_P, has been widely linked to the amelioration of metabolic outcomes in a context of obesity and other dysbiosis-associated diseases (Everard et al., 2013; Schneeberger et al., 2015). Herein, other taxonomic indicators were identified significantly driving this last cluster, such as *Lactobacillus*, *D. newyorkensis* and *Coriobacteriales_Insertae_sedis*, indicating that the CP-diet favor the growth of beneficial co-abundance taxa which may share functional adaptation upon the digestion of polyphenol-rich foods. In line with these observations, the CCA accounting for the taxonomic composition of mice gut microbiota provided insights into the association between the abundance of these genera and a lower BW in mice fed an obesogenic diet. Predicted KEGG categories such as energy metabolism, amino-acid and fatty acid metabolism that were dominant in HFHS-fed mice, as observed in previous study (Daniel et al., 2014), were repressed by polyphenol-rich diets. However, the accurate characterization of microbial functional pathways involved in the attenuation of metabolic disorders, require further studies by other precise methods such as transcriptomics, proteomics and metabolomics, useful for microbiome-based precision therapies. In terms of metabolic functions, the FRI explains why BF-fed mice kept the obesity phenotypes as observed in HFHS-fed mice. Bray–Curtis dissimilarities, while accounting for KEGG orthologs markers, indicate that the BF-group overlapped the HFHS, emphasizing that despite the fact that some taxa were significantly modulated by this fiber-rich diet, obesity-related metabolic functions were not influenced.

## Conclusion

The present study demonstrates that polyphenols from cranberry and not their constitutive fibers are involved in attenuating obesity-associated disturbances. Specifically, polyphenol-rich diets improved HFHS-induced gut microbiota dysbiosis. We highlight the selective prebiotic effects of polyphenols on *A. muciniphila*, *Muribaculaceae*, *D. newyorkensis*, *Angelakisella*, *Coriobacteriaceae*, and *Eggerthellaceae*. Among these taxa, *Eggerthellaceae*, and *Muribaculaceae* were revealed as key players related to BW loss and to a restored intestinal histomorphology. We highlight the prebiotic effects of cranberry and blueberry polyphenols on *D. newyorkensis*, a novel bacterial biomarker identified to counter metabolic diseases (Cox and Blaser, 2018).

Apart from the gut microbiota composition, assessment of its metabolic functional structure constitutes a relevant approach to understand the mechanism by which polyphenols can prevent metabolic disorders. Functional redundancy of the gut microbiota also emerges as a useful tool to study the bacteria metabolic contribution, and how specific taxa fulfill diversified functional roles in the host. Remarkably, cranberry powder strongly influenced energy efficiency, white adipose tissue reduction and BW loss in obese mice; these effects were linked to a shift in the gut microbiota enterotype and metabolic profile. The bloom of *Coriobacteriaceae* and *Eggerthellaceae* by fiber-rich CF, point to the presence of non-extractable polyphenols interacting with cranberry fibers; we infer that a synergy between cell wall polysaccharides and polyphenols in cranberry fiber-rich fraction ameliorates the liver TG and microbiota composition. Our findings support not only the use of polyphenols, but the whole berries containing polyphenols/cell wall polysaccharides, as preventative strategy against metabolic diseases by modulating the functional and compositional profile of the gut microbiota.

## Data Availability Statement

The datasets presented in this study can be found in online repositories. The names of the repository/repositories and accession number(s) can be found below: https://www.ncbi.nlm.nih.gov/bioproject/PRJNA628976.

## Ethics Statement

The animal study was reviewed and approved by animal care committee of Laval University (CPAUL). The protocols were summited and approved by Canadian Council on Animal Care.

## Author Contributions

YD, DR, GP, EL, and AM conceived the study. M-CR-D and MR were involved in the animal experiments. MR performed the metabolic assays. M-CR-D performed the histological analysis of colon tissues, performed the 16S rRNA sequences processing, analysis of the gut microbiota structure and functional profiling, and performed the statistical analysis of gut microbiome and mouse phenotypes. M-CR-D and YD discussed and wrote this manuscript. All authors contributed to reviewing and editing the manuscript.

## Conflict of Interest

The authors declare that the research was conducted in the absence of any commercial or financial relationships that could be construed as a potential conflict of interest.

## Footnotes

1 https://enterotype.embl.de/enterotypes.html
