# Supplementary material for: Berry Polyphenols and Fibers Modulate Distinct Microbial Metabolic Functions and Gut Microbiota Enterotype-Like Clustering in Obese Mice
Source: Front Microbiol. 2020 Aug 26;11:2032. doi: 10.3389/fmicb.2020.02032 (PMC7479096; doi:10.3389/fmicb.2020.02032)
Supplement: Supplementary file 1 [file Table_1.docx]

Supplementary Material

Table S1. Phenolic characterization of cranberry and blueberry dry powder

| Polyphenol content | Cranberry powder | Blueberry powder |
| --- | --- | --- |
| Total Anthocyanins (mg C3GE/100 g DW) | **138.7 ± 19.0** | **974.4 ± 103.8** |
| Delphinidin 3-galactoside | - | 92.8± 9.7 |
| Delphinidin 3-glucoside | - | 101.4± 10.5 |
| Cyanidin 3-galactoside | 39.1 ± 5.0 | 41.4 ± 3.9 |
| Delphinidin 3-arabinoside | - | 57.5 ± 6.0 |
| Cyanidin 3-glucoside | 1.6 ± 0.3 | 44.0 ± 4.5 |
| Petunidin 3-galactoside | - | 50.9 ± 5.3 |
| Cyanidin 3-arabinoside | 24.5 ± 3.5 | 25.9± 5.3 |
| Petunidin 3-glucoside | - | 75.8 ± 8.1 |
| Peonidin 3-galactoside | 51.0 ± 7.7 | 14.4 ± 1.4 |
| Petunidin 3-arabinoside | - | 278 ± 2.6 |
| Peonidin 3-glucoside | 4.9 ± 0.1 | 32.7 ± 33.6 |
| Malvidin 3-galactoside | - | 84.0 ± 12.2 |
| Malvidin 3-glucoside | - | 130.8 ± 13.7 |
| Peonidin 3-arabinoside | 17.7 ± 2.6 | - |
| Malvidin 3-arabinoside | - | 49.8 ± 4.5 |
| Delphinidin 3-(6"-acetoyl) glucoside | - | 30.0 ± 3.0 |
| Cyanidin 3-(6"-acetoyl) glucoside | - | 16.4 ± 2.9 |
| Malvidin 3-(6"-acetoyl) glucoside | - | 18.4 ± 3.1 |
| Petunidin 3-(6"-acetoyl) glucoside | - | 22.3 ± 3.4 |
| Peonidin 3-(6"-acetoyl) glucoside | - | 10.2 ± 0.9 |
| Malvidin 3-(6"-acetoyl) glucoside | - | 47.9 ± 5.9 |
| Total Proanthocyanins (mg EE/100 g DW) | **182.2 ± 10.9** | **574 ± 11.0** |
| Monomers | 16.3 ± 3.7 | 35.4 ± 4.6 |
| Dimers | 47.0 ± 3.3 | 55.2 ± 1.1 |
| Trimers | 15.2 ± 1.2 | 34.1 ± 0.3 |
| Tetramers | 9.6 ± 0.7 | 33.5 ± 0.8 |
| Pentamers | 5.1 ± 0.3 | 22.7 ± 0.3 |
| Hexamers | ND | 19.9 ± 0.3 |
| Heptamers | ND | 9.4 ± 0.4 |
| Octamers | ND | 6.9 ± 0.6 |
| Nonamers | ND | 6.6 ± 0.2 |
| Decamers | ND | 2.4 ± 0.7 |
| Polymers >10 | 88.9 ± 10.9 | 348.4 ± 7.1 |

Table S2. Chemical composition of cranberry and blueberry dry powders and fibrous fractions

| Composition (g/100) | Cranberry powder | Cranberry fibrous fraction | Blueberry powder | Blueberry fibrous fraction |
| --- | --- | --- | --- | --- |
| Protein | 1.4 | 9.0 | 2.5 | 10.4 |
| Carbohydrates | 58.6 | - | 76.8 | 5.3 |
| Total fibres | 25.1 | 65.4 | 15.4 | 68.3 |
| Lipids | 10.8 | 22.1 | 3.4 | 5.5 |
| Residual moisture | 2.1 | 2.0 | <0.5 | 6.0 |
| Ash | 2.0 | 4.6 | 1.9 | 4.5 |
| Energy (Kcal / 100g) | 437.01 | 484.11 | 409.14 | 385.84 |

Table S3. Proanthocyanidins (PACs) content in berry fibrous fractions

| PACs degree of polymerization (DP) | Cranberry fibrous fraction | | Blueberry fibrous fraction | |
| --- | --- | --- | --- | --- |
|  | **Extractible PACs** | **Non-extractible PACs** | **Extractible PACs** | **Non-extractible PACs** |
| Monomers | 0,24 ± 0,023 | 0,06 ± 0,002 | 0,10 ± 0,001 | 0 |
| Dimers | 0,38 ± 0,003 | 0 | 0,06 ± 0,003 | 0 |
| Trimers | 0,17 ± 0,007 | 0 | 0,06 ± 0,003 | 0 |
| Tetramers | 0,11 ± 0,004 | 0 | 0 | 0 |
| Pentamers | 0,07 ± 0,001 | 0 | 0 | 0 |
| Hexamers | 0,06 ± 0,002 | 0 | 0 | 0 |
| Heptamers | 0,04 ± 0,003 | 0 | 0 | 0 |
| Octamers | 0,02 ± 0,019 | 0 | 0 | 0 |
| Nonamers | 0 | 0 | 0 | 0 |
| Decamers | 0 | 0 | 0 | 0 |
| Polymers>10 | 0,13 ± 0,034 | 0 | 0,71 ± 0,113 | 0 |
| Total | **1,21 ± 0,095** | **0,06 ± 0,002** | **0,93 ± 0,12** | **0** |

Figure S1. Whole cranberry powder lowers body weight gain, body fat mass and energy efficiency in HFHS-induced obese mice. Mice were fed a HFHS-diet (HF), Chow (CT), polyphenol-rich whole cranberry powder (CP), cranberry fiber-rich fraction (CF), polyphenol-rich whole blueberry powder (BP) and blueberry fiber-rich fraction (BF) for 8 weeks. A) Weekly body weight; B) total body weight gain; C) Adipose tissue weights of mice at 8-week, D) Percentage of fat mass expressed relative to body weight at the time of sacrifice; E) Inguinal white adipose tissue (IWAT) weight; F) Epididymal white adipose tissues (EWAT) weight expressed relative to body weight at 8-week; G) Daily food intake; H) Energetic efficiency corresponding to the ratio of body weight to energy intake; I) Liver triglycerides level; J) Oral glucose tolerance test (GTT) at 7-week of treatment after a 12h fast; K) Area under the curve (AUC) during GTT; L) Plasma insulin at the same timepoint of GTT test; M) AUC of plasma insulin measurements during GTT; N) Changes in glycemia levels after 12 h fast; O) Changes in fasting plasma insulin levels during GTT; P) Insulin tolerance test (ITT) at 6-week of treatment after a 6 h fast. Q) AUC during ITT; R) Homeostatic model assessment of insulin resistance (HOMA-IR). Dotted lines correspond to the mean values of CT-fed mice. Values are expressed as mean ± SEM (n = 12). **p* < 0.05 ** *p*<0.01 *** *p*<0.001 and *****p*<0.0001 as compared to CT group. ##*p*<0.01 #*p*<0.05 compared to HFHS. p-values indicated in the graph “A” are compared to HF and colour-coded as the legend.


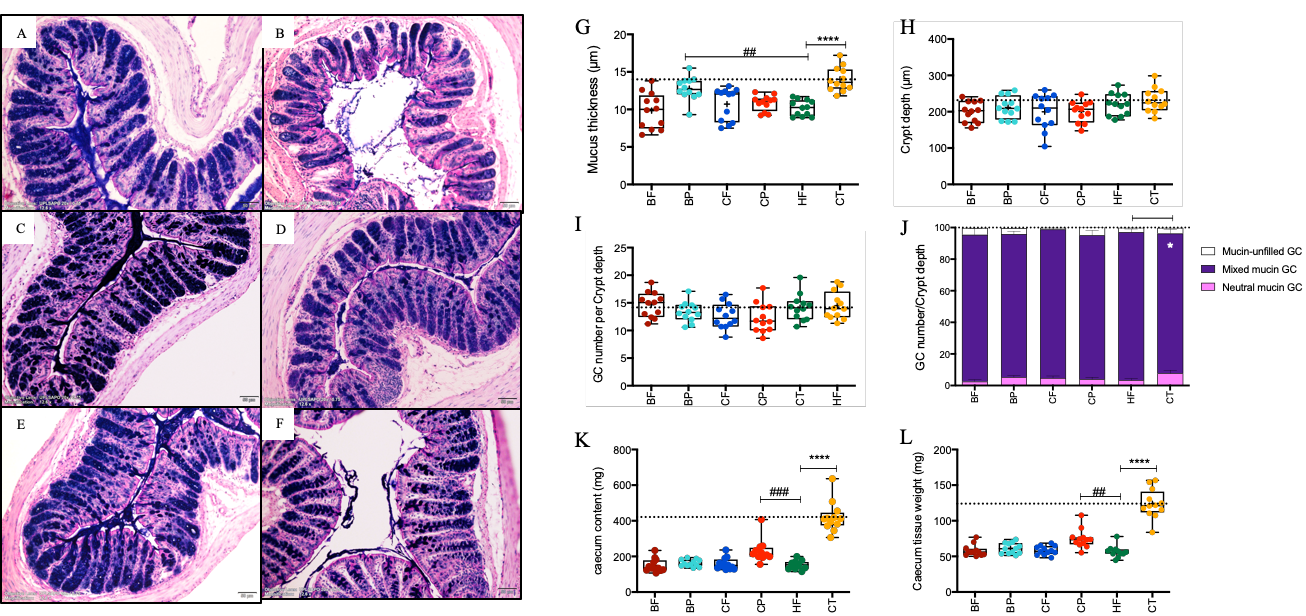


Figure S2. BP-diet improved the colonic mucus thickness while CP-diet increased the caecum size and mass in HFHS-induced obese mice. Representative histological images of the colon tissues of mice fed A) Chow (CT); B) HFHS-diet (HF); C) polyphenol-rich whole cranberry powder (CP); D) cranberry fiber-rich fraction (CF); E) polyphenol-rich whole blueberry powder (BP); and F) blueberry fiber-rich fraction (BF). A combination of Alcian Blue and periodic acid–Schiff staining (AB/PAS) was used to distinguish acidic (dark blue) and neutral (red) mucins. Purple colour indicates both acidic and neutral mucins. Images were taken using objective lens UPLSAPO 20X/0.75, magnification 12.6X, Scale 50 μm. Histological parameters were evaluated in cross-sections of colon tissues stained with AB/PAS staining: G) Mucus thickness, H) Crypt depth I) Total goblet cells (GC) number per crypt µm, and J) Total mucin-filled GC number. The weight of K) caeca content and L) tissues were registered as indicative of the impact of diets on the microbial community changes. Data are shown as mean ± SEM (n = 12/group). Significant differences were determined by ordinary one-way ANOVA. Values are expressed as mean +/- SEM. *****p*<0.0001, ***p*<0.01 and **p*<0.05 compared to HFHS. Chow group is represented by the dotted line.

Figure S3. Polyphenol-rich CP and BP diets reduced the relative proportions of gut opportunistic bacteria and triggered health-promoting taxa in HFHS-induced obese mice. Polyphenol-rich CP and BP significantly decreased the relative abundance of phylum *Firmicutes* and selectively increased *Verrrucomicrobia* and *Actinobacteria* phyla in HFHS-induced obese mice. A) Bar graph shows changes in the relative abundance of gut microbiota phyla at 8-week in mice fed HFHS (HF), Chow (CT), HFHS-diet supplemented with either polyphenol-rich whole cranberry powder (CP), cranberry fiber-rich fraction (CF), polyphenol-rich whole blueberry powder (BP) and blueberry fiber-rich fraction (BF); B) Bar graph of the relative abundances of bacterial families across the gut microbiota composition of each group; C) Bar graph of the relative abundances of genera composing the gut microbiota of each group; D) Correlation between the relative abundance of *A. muciniphila* analyzed by 16S rRNA gene sequencing and by qPCR; each point in a plot represents values of the linear combinations for a sample pair in the correlation (n=12/group). Spearman correlation R^2^ scores are indicated in the graphs. Asterisks indicate taxa for which the association was significant, **p*<0.05, ***p*<0.01, ****p*<0.001, *****p*<0.0001. E) Absolute quantification of 16S rRNA gene copy of *A. muciniphila* per gr of fecal samples. Kruskal-Wallis test with FDR Benjamini and Hochberg post-hoc multiple comparison correction was performed to compare taxonomic abundance among groups. #### *p*<0.0001, ### *p*<0.001 as compared to CT-group and **p*<0.05, ***p*<0.01, ****p*<0.001, *****p*<0.0001 as compared to HFHS.

Figure S4. Enrichment of signature taxa in enterotype-like clusters of mice gut microbiota. Relative abundances of taxa indicator of enterotype-like clusters of mice fed a Chow-diet or a HFHS-diet supplemented either with polyphenol-rich berry powders or their fiber-rich fractions. Three enterotypes were identified: ET_1 (*Bacteroidetes/Muribaculaceae*); ET_2 (*Prevotella/Akkermansiaceae*) and ET_3 (*Firmicutes/Ruminococcus*). Line inside the box represents the median, the mark represents the mean, while whiskers represent the lowest and highest values within 1.5 interquartile range (IQR).


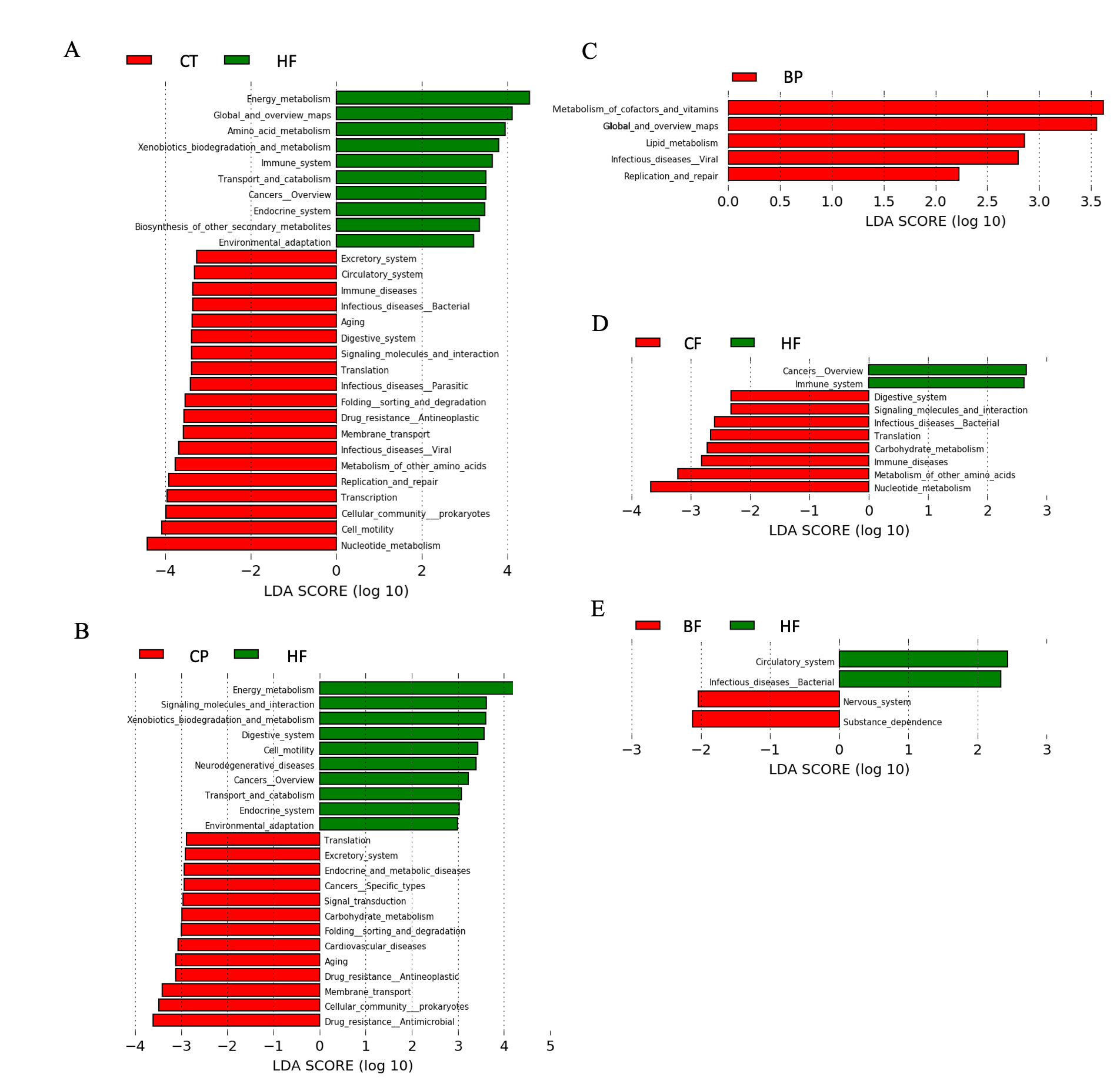


Figure S5. Key predicted level 2 KEGG functional pathways differently enriched in mice gut microbiota. Level 2 KEGG pathways characterizing the microbial functional composition were identified using linear discriminant analysis (LDA) combined with effect size (LEfSe) algorithm. A) Predicted microbial function capacities enriched in the mice fed HFHS-diet (HF) as compared to Chow (CT); B) Microbial functional pathways enriched in mice fed polyphenol-rich whole cranberry powder (CP) as compared to HF; C) Microbial functional pathways enriched in mice fed polyphenol-rich whole blueberry powder (BP) as compared to HF; D) Microbial functional pathways enriched in mice fed cranberry fiber-rich fraction as compared to HF (CF); and E) Microbial functional pathways enriched in mice fed blueberry fiber-rich fraction (BF) as compared to HF. Threshold for LDA scores was 2.0. Positive LDA (green bars) are enriched in HF, while negative LDA (red bars) are enriched in CT, CP, BP, CF and BF as shown on figure legends (n=12/group). Statistically significant taxa enrichment among groups was obtained with Kruskal-Wallis test among classes (*p*-value = 0.05).

Figure S6. Level 1 KEGG pathways distinguishing the functional composition of the gut microbiota of mice fed the HFHS-diet and the Chow-diet. Level 1 KEGG pathways characterizing the microbial functional composition were identified using linear discriminant analysis (LDA) combined with effect size (LEfSe) algorithm. Threshold for LDA scores was 2.0. Positive LDA (green bars) are enriched in HFHS-fed mice (HF), while negative LDA (red bars) are enriched in Chow-fed mice (CT) as shown on figure legends. Statistically significant taxa enrichment among groups was obtained with Kruskal-Wallis test among classes (*p* value = 0.05).

Figure S7. Level 1 KEGG pathways significantly differentiating the functional composition of the gut microbiota of mice fed polyphenol-rich CP and BP and their fiber-rich fractions CF and BF as compared to HFHS-fed mice. Level 1 KEGG pathways distinguishing the functional profile of the gut microbiota were identified using linear discriminant analysis (LDA) combined with effect size (LEfSe) algorithm. A) Microbial functional pathways enriched in mice fed polyphenol-rich whole cranberry powder (CP) as compared to HFHS (HF); B) Microbial functional pathways enriched in mice fed polyphenol-rich whole blueberry powder (BP) as compared to HF; C) Microbial functional pathways enriched in mice fed cranberry fiber-rich fraction as compared to HF (CF); and D) Microbial functional pathways enriched in mice fed blueberry fiber-rich fraction (BF) as compared to HF. Threshold for LDA scores was 2.0. Positive LDA (green bars) are enriched in HF, while negative LDA (red bars) are enriched in CP, BP, CF and BF as shown on figure legends (n=12/group). Statistically significant taxa enrichment among groups was obtained with Kruskal-Wallis test among classes (*p*-value = 0.05).

Table S4. KEGG microbial functions significantly correlated with changes in mouse body weight as determined by MaAsLin multivariate model

| Variable | Microbial Functional Feature | Coefficient | N | N.not.0 | *p*-value | *q*-value |
| --- | --- | --- | --- | --- | --- | --- |
| Body_weight | Phosphotransferase system (PTS) | -0.0059225 | 72 | 72 | 7.49E-05 | 0.00292188 |
| Body_weight | Amino sugar and nucleotide sugar metabolism | -0.0020872 | 72 | 72 | 0.00042764 | 0.00902506 |
| Body_weight | Fructose and mannose metabolism | -0.0013018 | 72 | 72 | 0.00076182 | 0.01313482 |
| Body_weight | Glutathione metabolism | -0.0011398 | 72 | 72 | 0.00019422 | 0.00571234 |
| Body_weight | Primary bile acid biosynthesis | -0.0008386 | 72 | 72 | 0.00085033 | 0.01400381 |
| Body_weight | DNA replication | -0.0008198 | 72 | 72 | 5.24E-05 | 0.00290966 |
| Body_weight | Pentose phosphate pathway | -0.0007384 | 72 | 72 | 5.48E-06 | 0.00161805 |
| Body_weight | Riboflavin metabolism | -0.0006695 | 72 | 72 | 0.00129273 | 0.0191842 |
| Body_weight | Prolactin signaling pathway | -0.0006553 | 72 | 72 | 0.0015496 | 0.02213708 |
| Body_weight | Penicillin and cephalosporin biosynthesis | -0.0006482 | 72 | 72 | 0.00130453 | 0.0191842 |
| Body_weight | Cyanoamino acid metabolism | -0.0006368 | 72 | 72 | 1.91E-05 | 0.00161805 |
| Body_weight | Homologous recombination | -0.0005034 | 72 | 72 | 0.00061627 | 0.01100476 |
| Body_weight | Phenylpropanoid biosynthesis | -0.0004368 | 72 | 72 | 0.00048596 | 0.00913766 |
| Body_weight | Peptidoglycan biosynthesis | -0.0003806 | 72 | 72 | 0.00046376 | 0.00913766 |
| Body_weight | RNA polymerase | -0.0003797 | 72 | 72 | 0.00031094 | 0.00706675 |
| Body_weight | Proteasome | -0.0002985 | 72 | 60 | 0.00259651 | 0.03606261 |
| Body_weight | Type II diabetes mellitus | -0.0001315 | 72 | 72 | 2.27E-05 | 0.00161805 |
| Body_weight | Choline metabolism in cancer | 0.00042113 | 72 | 72 | 0.00010017 | 0.00357748 |
| Body_weight | Vitamin B6 metabolism | 0.00060283 | 72 | 72 | 0.0004332 | 0.00902506 |
| Body_weight | Novobiocin biosynthesis | 0.00062698 | 72 | 72 | 0.00015897 | 0.00496791 |
| Body_weight | Valine, leucine and isoleucine biosynthesis | 0.00091862 | 72 | 72 | 0.00086824 | 0.01400381 |
| Body_weight | Phosphonate and phosphinate metabolism | 0.00092898 | 72 | 72 | 1.62E-05 | 0.00161805 |
| Body_weight | 2-Oxocarboxylic acid metabolism | 0.00111864 | 72 | 72 | 0.00028409 | 0.00676395 |
| Body_weight | Oxidative phosphorylation | 0.00115897 | 72 | 72 | 7.60E-05 | 0.00292188 |
| Body_weight | Glyoxylate and dicarboxylate metabolism | 0.00118521 | 72 | 72 | 0.00024553 | 0.00641461 |
| Body_weight | Biosynthesis of antibiotics | 0.00146004 | 72 | 72 | 2.14E-05 | 0.00161805 |
| Body_weight | Biosynthesis of amino acids | 0.00150048 | 72 | 72 | 0.00013508 | 0.00450258 |
| Body_weight | Phenylalanine, tyrosine and tryptophan biosynthesis | 0.00152835 | 72 | 72 | 0.00049343 | 0.00913766 |
| Body_weight | Biosynthesis of secondary metabolites | 0.00170713 | 72 | 72 | 0.00023538 | 0.00641461 |
| Body_weight | Citrate cycle (TCA cycle) | 0.00174812 | 72 | 72 | 7.50E-05 | 0.00292188 |

Table S5. MaAsLin significant correlations between KEGG microbial functions and energy efficiency and liver triglycerides level in mice.

| Variable | Microbial Functional Feature | Coefficient | N | N.not.0 | *p*-value | *q*-value |
| --- | --- | --- | --- | --- | --- | --- |
| Energy_efficiency | Phosphotransferase system (PTS) | -4.3164784 | 72 | 72 | 0.00013883 | 0.00079608 |
| Energy_efficiency | ABC transporters | -2.8608865 | 72 | 72 | 3.82E-05 | 0.0005455 |
| Energy_efficiency | Starch and sucrose metabolism | -2.5602155 | 72 | 72 | 8.51E-05 | 0.00079003 |
| Energy_efficiency | Glycolysis / Gluconeogenesis | -1.2523014 | 72 | 72 | 3.38E-05 | 0.0005455 |
| Energy_efficiency | Glutathione metabolism | -0.8400877 | 72 | 72 | 0.00028179 | 0.00136617 |
| Energy_efficiency | Pyrimidine metabolism | -0.7761125 | 72 | 72 | 3.87E-05 | 0.0005455 |
| Energy_efficiency | Mismatch repair | -0.7462071 | 72 | 72 | 3.93E-05 | 0.0005455 |
| Energy_efficiency | Degradation of aromatic compounds | -0.716631 | 72 | 72 | 3.68E-05 | 0.0005455 |
| Energy_efficiency | cGMP-PKG signaling pathway | -0.7095958 | 72 | 72 | 0.00013102 | 0.00079003 |
| Energy_efficiency | cAMP signaling pathway | -0.7095958 | 72 | 72 | 0.00013102 | 0.00079003 |
| Energy_efficiency | Cardiac muscle contraction | -0.7095958 | 72 | 72 | 0.00013102 | 0.00079003 |
| Energy_efficiency | Adrenergic signaling in cardiomyocytes | -0.7095958 | 72 | 72 | 0.00013102 | 0.00079003 |
| Energy_efficiency | Insulin secretion | -0.7095958 | 72 | 72 | 0.00013102 | 0.00079003 |
| Energy_efficiency | Aldosterone synthesis and secretion | -0.7095958 | 72 | 72 | 0.00013102 | 0.00079003 |
| Energy_efficiency | Aldosterone-regulated sodium reabsorption | -0.7095958 | 72 | 72 | 0.00013102 | 0.00079003 |
| Energy_efficiency | Endocrine and other factor-regulated calcium reabsorption | -0.7095958 | 72 | 72 | 0.00013102 | 0.00079003 |
| Energy_efficiency | Gastric acid secretion | -0.7095958 | 72 | 72 | 0.00013102 | 0.00079003 |
| Energy_efficiency | Pancreatic secretion | -0.7095958 | 72 | 72 | 0.00013102 | 0.00079003 |
| Energy_efficiency | Protein digestion and absorption | -0.7030792 | 72 | 72 | 5.87E-05 | 0.0006376 |
| Energy_efficiency | Bile secretion | -0.6918516 | 72 | 72 | 0.00016622 | 0.00090336 |
| Energy_efficiency | Mineral absorption | -0.6725455 | 72 | 72 | 9.75E-05 | 0.00079003 |
| Energy_efficiency | Chloroalkane and chloroalkene degradation | -0.6582353 | 72 | 72 | 9.29E-06 | 0.0005455 |
| Energy_efficiency | Primary immunodeficiency | -0.6458242 | 72 | 72 | 0.00012589 | 0.00079003 |
| Energy_efficiency | Glycerolipid metabolism | -0.6306738 | 72 | 72 | 0.00012953 | 0.00079003 |
| Energy_efficiency | Naphthalene degradation | -0.6204404 | 72 | 72 | 3.73E-05 | 0.0005455 |
| Energy_efficiency | DNA replication | -0.6050003 | 72 | 72 | 7.96E-05 | 0.00079003 |
| Energy_efficiency | Pentose phosphate pathway | -0.5481787 | 72 | 72 | 8.15E-06 | 0.0005455 |
| Energy_efficiency | Cyanoamino acid metabolism | -0.4711767 | 72 | 72 | 2.89E-05 | 0.0005455 |
| Energy_efficiency | Carbon fixation in photosynthetic organisms | -0.4094607 | 72 | 72 | 4.37E-05 | 0.00055539 |
| Energy_efficiency | Drug metabolism - other enzymes | -0.3110524 | 72 | 72 | 9.40E-05 | 0.00079003 |
| Energy_efficiency | Ribosome biogenesis in eukaryotes | -0.2547038 | 72 | 72 | 0.00014011 | 0.00079608 |
| Energy_efficiency | Vancomycin resistance | -0.2472966 | 72 | 72 | 0.00013273 | 0.00079003 |
| Energy_efficiency | Type II diabetes mellitus | -0.0987267 | 72 | 72 | 2.52E-05 | 0.0005455 |
| Energy_efficiency | Peroxisome | 0.23960372 | 72 | 72 | 0.0001501 | 0.00083389 |
| Energy_efficiency | Choline metabolism in cancer | 0.31476786 | 72 | 72 | 0.00011818 | 0.00079003 |
| Energy_efficiency | Ubiquinone and other terpenoid-quinone biosynthesis | 0.47828201 | 72 | 72 | 0.00021295 | 0.00111461 |
| Energy_efficiency | Selenocompound metabolism | 0.55908577 | 72 | 72 | 0.00021401 | 0.00111461 |
| Energy_efficiency | Phosphonate and phosphinate metabolism | 0.69850007 | 72 | 72 | 1.74E-05 | 0.0005455 |
| Energy_efficiency | Folate biosynthesis | 0.71703049 | 72 | 72 | 3.92E-05 | 0.0005455 |
| Energy_efficiency | Arginine biosynthesis | 0.81467295 | 72 | 72 | 1.98E-05 | 0.0005455 |
| Energy_efficiency | Biotin metabolism | 0.84148802 | 72 | 72 | 1.90E-05 | 0.0005455 |
| Energy_efficiency | Nitrotoluene degradation | 0.86780223 | 72 | 72 | 2.07E-05 | 0.0005455 |
| Energy_efficiency | Oxidative phosphorylation | 0.89092059 | 72 | 72 | 5.32E-05 | 0.00061504 |
| Energy_efficiency | Biosynthesis of antibiotics | 1.06334308 | 72 | 72 | 4.44E-05 | 0.00055539 |
| Energy_efficiency | Biosynthesis of amino acids | 1.08551728 | 72 | 72 | 0.00026873 | 0.00136617 |
| Energy_efficiency | Citrate cycle (TCA cycle) | 1.34168133 | 72 | 72 | 5.41E-05 | 0.00061504 |
| Liver_TG | Degradation of aromatic compounds | -4.75E-05 | 72 | 72 | 3.89E-05 | 0.00353277 |
| Liver_TG | Ethylbenzene degradation | -4.56E-05 | 72 | 72 | 3.30E-06 | 0.00058828 |
| Liver_TG | alpha-Linolenic acid metabolism | -4.53E-05 | 72 | 72 | 3.53E-06 | 0.00058828 |
| Liver_TG | Quorum sensing | -4.40E-05 | 72 | 72 | 0.00010669 | 0.0088905 |
| Liver_TG | Naphthalene degradation | -4.35E-05 | 72 | 72 | 1.18E-05 | 0.00131009 |
| Liver_TG | Pentose phosphate pathway | -3.79E-05 | 72 | 72 | 3.06E-06 | 0.00058828 |
| Liver_TG | Vancomycin resistance | -1.83E-05 | 72 | 72 | 1.56E-05 | 0.00155561 |
| Liver_TG | Folate biosynthesis | 5.07E-05 | 72 | 72 | 1.04E-05 | 0.00129991 |
| Liver_TG | Biosynthesis of antibiotics | 7.83E-05 | 72 | 72 | 4.51E-06 | 0.00064361 |
